# The relationship between early life course air pollution exposure and general health in adolescence in the United Kingdom

**DOI:** 10.1038/s41598-025-94107-w

**Published:** 2025-05-14

**Authors:** Gergő Baranyi, Katie Harron, Youchen Shen, Kees de Hoogh, Emla Fitzsimons

**Affiliations:** 1 Centre for Longitudinal Studies, UCL Institute of Education, University College London, London, UK; 2 Population, Policy and Practice Department, UCL GOS Institute of Child Health, University College London, London, UK; 3 Institute for Risk Assessment Sciences, Utrecht University, Utrecht, The Netherlands; 4 Swiss Tropical and Public Health Institute, Allschwil, Switzerland; 5 University of Basel, Basel, Switzerland

**Keywords:** Air pollution, Fine particle, General health, Life course, Environmental inequality, Birth cohort, Environmental impact, Paediatric research

## Abstract

Air pollution is associated with health in childhood. However, there is limited evidence on sensitive periods during the first 18 years of life. Data were drawn from the Millennium Cohort Study, a large and nationally representative cohort born in 2000/2002. Self-reported general health was assessed at age 17; number of hospital records were derived from linked health data (Hospital Episode Statistics) for consented participants. Residential history was linked to 25 × 25 m grid resolution annual PM_2.5_, PM_10_ and NO_2_ maps between 2000 and 2019; year-specific air pollution exposure in 200-m buffers around postcode centroids were computed. After adjusting for individual and time-variant area-level confounders, children exposed to higher air pollution in early (2–4 y) (*n* = 9137; PM_2.5_: OR = 1.06, 95% CI: 1.01–1.11; PM_10_: OR = 1.05, 95% CI: 1.01–1.09; NO_2_: OR = 1.01, 95% CI: 1.00–1.02) and middle childhood (5–7) (*n* = 9171; PM_2.5_: OR = 1.04, 95% CI: 1.00–1.07; PM_10_: OR = 1.03, 95% CI: 1.01–1.06) reported worse general health at age 17. Higher PM_2.5_ and NO_2_ exposure in adolescence increased the number of hospital episodes in young adulthood. Individuals from non-White and disadvantaged backgrounds were exposed to higher levels of air pollution. Air pollution in early and middle childhood might contribute to worse general health, with ethnic minority and disadvantaged children being more exposed.

**Supplementary Information:**

The online version contains supplementary material available at 10.1038/s41598-025-94107-w.

## Introduction

Poor air quality is the single biggest environmental threat to human health, and according to the World Health Organization, 99% of the global population lives in areas exceeding healthy thresholds^1^. Based on the Global Burden of Diseases 2021 estimation, air pollution is the second leading cause of mortality responsible to 8.1 million death globally (12% of total); the diseases burden is particularly high among children and in older age^2^. Early life air pollution exposure may influence birth outcomes^3^. Morphological and functional development of organ systems (e.g. respiratory health, neurodevelopment) in early years makes children more vulnerable to toxic air quality^4^: emerging research shows higher risk of respiratory diseases^5^, such as asthma^6^, high blood pressure^7^, obesity^8^, mental health problems^9^ and lower cognitive function^10^ among children exposed to higher air pollution. The long-term health effects are also notable; the impact of early life air pollution might be mediated via childhood health, cognition and socioeconomic status on later life health outcomes^11^ and ultimately on mortality^12^.

Applying the life-course approach^13^ in the environmental health literature is crucial to understand whether there are sensitive developmental periods during childhood where the detrimental impact of air pollution is severe and long lasting^4^, or the negative effect of bad air quality accumulates over time with longer exposure leading to worse health. Evidence is limited as this requires cohort studies with address history allowing to link time-varying air pollution estimates over a long period of time^9,14–18^. Controlling for potential time-varying area-level confounders (e.g. income deprivation, population density, noise) is a further limitation, as well as sufficient resolution of air pollutants to produce rigorous evidence^19^. Air pollution exposure is often higher in areas with disadvantaged individuals^20^, and among ethnic minorities^21^ with potential effect modifications^22^; representative studies are required to estimate unbiased pollution-health associations and investigate environmental inequalities.

Using a large and nationally representative British birth cohort, we explored the association between exposure to air pollution from birth onwards and general health at age 17; with outcomes measured using both self-reported and administrative health records. The relationships were investigated with three pollutants relevant to population health: fine particulate matter that are 2.5 μm or less in diameter (PM_2.5_), particulate matter with a diameter of 10 μm or less (PM_10_), and nitrogen dioxide (NO_2_). First, considering six life-course models (i.e., infancy, early childhood, middle childhood, late childhood, adolescence, accumulation), associations were estimated with general health. Second, average life-course exposure between sociodemographic groups, as well as effect modification by sex, ethnicity, parental education and area-deprivation were explored.

## Methods

The Millennium Cohort Study (MCS) is a nationally representative cohort of children born between September 2000 and January 2002 in the United Kingdom^23^. Households with a child aged 9 months of age (Sweep 1) and living in the UK were randomly selected, providing a nationally representative sample^23^. Smaller UK nations, and in England disadvantaged areas, and families with ethnic minority backgrounds were oversampled, and at age 3 (Sweep 2), further eligible children—not captured at baseline—were added to the sample. Participants have been followed up seven times to date: at the average age of 9 months (Sweep 1: 2000–2002), 3 years (Sweep 2: 2003–2005), 5 years (Sweep 3: 2006), 7 years (Sweep 4: 2008), 11 years (Sweep 5: 2012–2013), 14 years (Sweep 6: 2015–2016), and 17 years (Sweep 7: 2017–2019). The total sample size comprises 19,519 children within 19,244 families ever interviewed^24^. In the analytical sample, we included children if they participated in the age 17 sweep and did not have missing values: data on self-reported general health covered all four UK nations; hospital episodes were only available for England. All methods were carried out in accordance with relevant guidelines and regulations and informed consent was obtain from all subjects.

### Exposure to air pollution

Annual average PM_2.5_, PM_10_, and NO_2_ were estimated for Europe using Geographically and Temporally Weighted Regression (GTWR) between 2000 and 2019 on a 25 × 25 m grid. Detailed descriptions of the model development can be found in Shen et al.^25^. Briefly, the GTWR models regressed annual average observations from routine monitoring stations on several spatial predictor variables, such as chemical transport model estimates, satellite-derived data, meteorological data, and land-use and road variables, capturing spatio-temporal variations in the measured annual average air pollution levels. GTWR allowed the regression coefficients to vary in space and time, reflecting the changing dynamics of air pollution across Europe over a 20-year period. The GTWR models showed satisfactory performance, explaining 71–82% of the variance in PM_2.5_ levels, 50–68% of the variance in PM_10_ levels and 61–69% of the variance in NO_2_ levels, as given by 5-fold cross-validated R^2^ values^25^.

Residential postcodes at interviews—as well as at birth for those moving between birth and sweep 1—were geocoded into 1-m resolution grid reference (i.e. easting [X], northing [Y]) using the British National Grid (England, Scotland, and Wales) and the Irish Grid (Northern Ireland) coordinate systems. Postcodes are the smallest geographic units in the UK, there are approximately 1.8 million live postcodes with an average of 18 households each. Monthly residential postcode history was created using information about the dates of birth, dates of interviews, and residential moves. If the interview date was missing despite a productive interview, country-specific median dates were imputed. Main respondents were asked in each sweep whether their address had changed since the last interview, and if so, when they moved to their current address; to avoid discrepancies, we considered move dates valid if they took place between two productive interviews.

Average residential air pollution exposure for each year of participants’ lives were derived using buffers around postcode centroids. Monthly postcode history was linked to annual air pollution concentrations and averaged for every 12 months from birth onwards. Instead of extracting the pollution concentration at the grid reference, we computed 100, 200, and 500-m buffers around postcode centroids and extracted the area-weighted average pollution concentration within buffers. Using buffers aimed to lower exposure misclassification due to distance error between postcode and property centroids. As previous internal CLS analyses on MCS COVID web survey data suggested that the mean distance error between postcode and residential centroid is 60 m (SD = 87 m)^26^, we chose 200-m buffers as the main analyses as it likely covered at least 95% of the residential properties. Finally, we computed mean exposure during infancy (0–1 years), early childhood (2–4 years), middle childhood (5–7 years), late childhood (8–11 years), and adolescence (from 12 years until the interview in Sweep 7 [~ 17 years]), as well as during participants’ whole life (i.e., accumulated exposure, 0–17 years); classification aligns with schooling milestones in the UK (i.e. pre-school, key stage 1–4). Supplementary Fig. 1 suggested that the exposure specific correlations across life years, as well as correlations between developmental period-specific PM_2.5_, PM_10_ and NO_2_ exposures were very high among participants.

### Outcomes

General health was measured using a self-reported and a health administrative data derived variable.

### Self-reported general health

MCS participants at Sweep 7 (average age of 17) were asked how they would describe their health generally, with five response options provided (‘Excellent’; ‘Very Good’; ‘Good’; ‘Fair’; and ‘Poor’). As there were very few individuals with ‘Poor’ and ‘Fair’ general health, we merged these groups together.

### Number of hospital episodes

At Sweep 7, participants were asked to provide written consent linking their health records to the survey data^27^, and 85% of the sample provided consent. For MCS participants residing in England, extraction from the Hospital Episode Statistics (HES) database was carried out by National Health Service (NHS) England using information on name, sex, date of birth, and postcode^27,28^. The match rate was 81.5%; however, for participants not linked to HES records we assumed the absence of hospital attendances, rather than the failure of linkage processes^29^. Hospital episodes (i.e., continuous period of care under one consultant) were extracted from the Admitted Patient Care (HES-APC) dataset which covers almost the entire study period for consented and linked participants (01.01.2001–31.03.2020) and provides information about emergency and non-emergency admissions to secondary care requiring a hospital bed^30^. HES-APC has an almost universal coverage with 99% of hospital activity in England being funded by NHS^30^. We derived the number of hospital episodes from early childhood (2 year), middle childhood (5 year), late childhood (8 year), adolescence (12 year) and young adulthood (18 year) onwards as outcomes.

### Covariates

**Fig. 1:**
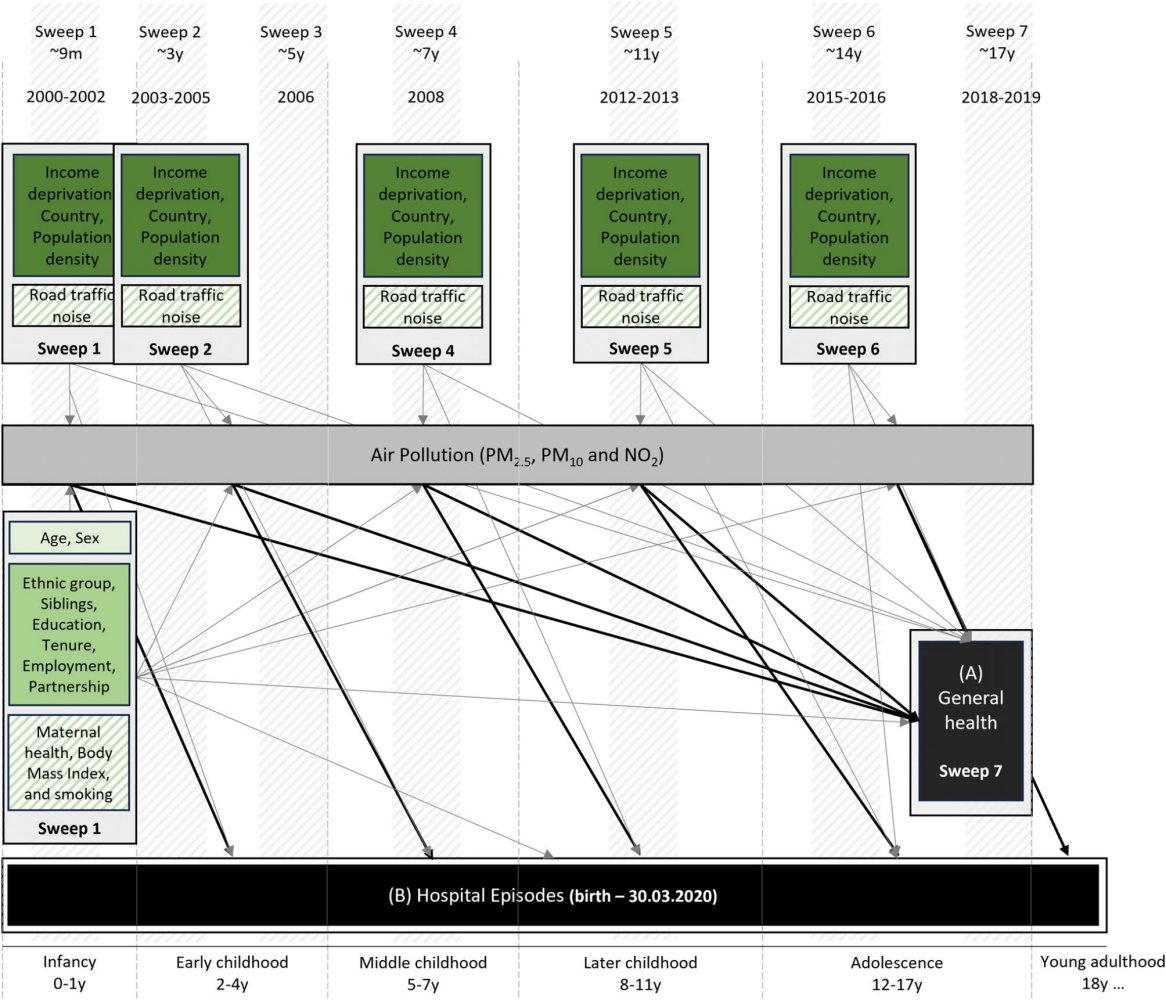
Graph showing assessed life-course associations between air pollution and general health. Black arrows are associations of interest, grey arrows are confounding pathways. Light-green shaded box includes Model 1, medium-green box Model 2, and dark-green box Model 3 confounders; boxes with diagonal stripes are sensitivity analyses. Data collection sweeps and developmental periods are also shown.

Confounders were selected using a directed acyclic graph, taking into consideration the time of exposures (Fig. 1). Individual-level covariates were time-invariant and derived at 9 months; if not available, at age 3. They included sex (male; female), ethnic groups (white; non-white [specific ethnic groups were merged to allow testing effect modification]), number of siblings (none; 1; 2 or more), parental partnership status (single parent; living with partner), household employment, household tenure, and highest household education. Household employment was derived from the main caregiver and their partners employment status (both employed; one employed; none employed), for household tenure ‘own’ (outright, with mortgage/loan, shared equity), ‘social rent’, ‘private rent’, and ‘other’ (e.g. living with parents, squatting) options were considered. Highest household education was defined based on the National Vocational Qualification (NVQ) scale: the highest level between the main caregiver and their partner was taken and merged into four groups: ‘None or overseas only’, ‘NVQ 1–2’, ‘NVQ 3’ and ‘NVQ 4–5’. We extracted age (in years) at Sweep 7 to account for small differences in the time of outcome data collection.

Area-level confounders at the time of air pollution exposure were entered in the models as time-variant. They were linked to sweep addresses, and included country (England, Northern Ireland, Scotland, and Wales), population density and income deprivation (see Supplementary Table 1 for more detail). Population density and income deprivation was measured across small areas: lower layer super output areas for England and Wales (1000 to 3000 persons), data zones for Scotland (500 to 1000 persons), and super output areas for Northern Ireland (1300 to 2800 persons). Population density (population counts per hectare) was derived from the 2001 and 2011 Census estimates. Small area-level relative deprivation is published independently across the four nations as part of English, Northern Irish, Scottish and Welsh indices of multiple deprivation; the income deprivation domain was selected as most consistently measured across nations and time. Country-specific income deprivation ranks were classified into four equal categories ranging from ‘Q1—Most deprived’ to ‘Q4—Least deprived’.

### Statistical analysis

Analyses for self-reported general health were conducted using proportional odds models, considering the stepwise increasing ordinal nature of the outcome. For the number of hospital episodes, we conducted quasi-Poisson regression to avoid overdispersion. MCS was designed to be representative of the UK population: to account for oversampling as well as to restore the representativeness we applied UK-wide (self-reported general health) and England-only (hospital episodes) complex survey weights in all presented analyses, including descriptive statistics. Analytical samples were separately defined for each exposure period to avoid restriction to children participating in all sweeps; analyses were run separately for each air pollutant.

In Model 1, we adjusted for age and sex. In Model 2, all individual-level variables were added (i.e., age, sex, ethnic groups, number of siblings, partnership status, highest household education, household tenure, and household employment). Finally, in Model 3, we additionally included population density, area income deprivation, as well as country at the time of exposure. Odds Ratios (ORs) or Incidence Rate Ratios (IRRs) and their 95% Confidence Intervals (95% CI) are reported per 1-µg m^−3^ increment; we also provide coefficients expressed as interquartile range (IQR) change. For self-reported general health at age 17, we run a post-hoc analysis estimating the associations for each year of exposure, and plotted ORs with smoothed curves using LOESS regression. Effect modification by sex, ethnic groups, highest household education and area-level income deprivation at the time of exposure was tested by adding an interaction term to Model 3. As we tested a very large number of interactions (i.e. by three pollutants and two outcomes), only false discovery rate (FDR) adjusted significant (*p*_*FDR*_) findings are interpreted to lower the risk of false positives due to multiple comparisons^31^.

Six sensitivity and supplementary analyses were carried out to test the robustness of our findings. First, we presented main results after deriving air pollution exposure using 100-m and 500-m buffers (*S1*). Second, fully-adjusted models were further adjusted for covariates about mother’s health status: self-reported general health (excellent; good; fair; poor), Body Mass Index before birth (underweight [< 18.5]; normal weight [18.5–24.9]; overweight [25.0–29.9]; obese [≥ 30]), and smoking status (never smoked; smoked during pregnancy; reduced or quit smoking before pregnancy; smoked before or after pregnancy) (*S2*). Third, road traffic noise (L_den_) was added to the final models: we downloaded the 2012 noise maps separately for each nations for (a) major roads (≥ 3,000,000 vehicles per year) and (b) road noise in agglomerations (≥ 100,000 residents). Maps were intersected with residential postcode centroids and extracted values were classified into 3 groups (≤ 54.9 decibel [dB]; 55.00–59.9 dB; ≥ 60 dB) (*S3*). Fourth, to account for bias due to missing individual and area-level confounders (8–9%) we ran multiple imputations on 25 datasets and combined coefficients using Rubin’s rule. In addition to all study variables, auxiliary variables were added including income quartiles, longstanding illness, maternal diabetes, sweep when family entered the study (sweep 1 or sweep 2), governmental region, as well as time-varying urban/rural classification and overall deprivation (*S4*). Fifth, one of the main underlying assumptions of proportional odds regression is proportional effects of exposure on outcome thresholds, thus we also provided fully adjusted models for self-reported general health using multinomial regression (*S5*). Finally, as two-pollutant models were not feasible given high multicollinearity, we provided estimates for PM_10_, PM_2.5_ and NO_2_ mixtures using quantile g-computation for multinomial outcomes^32^ for self-reported general health (*S6*).

Analyses were conducted in R version 4.3.0^33^, using the survey^34^, svyVGAM^35^, and mice^36^ packages.

## Results

**Fig. 2:**
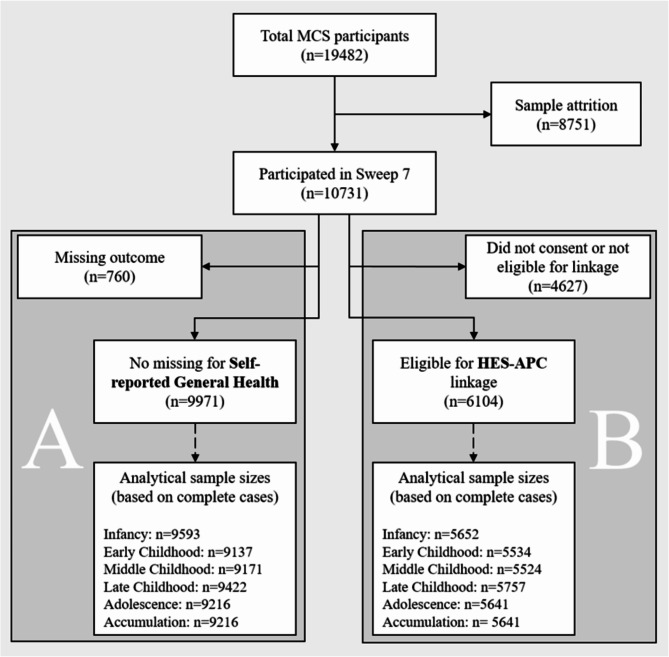
Flowchart presenting sample selection for self-reported general health (**A**) and hospital episodes (**B**).

Out of 10,731 individuals participating in the age 17 data collection, 9971 had no missing value for self-reported general health covering all UK nations (i.e. England, Northern Ireland, Scotland, Wales). From the English subsample, 6104 individuals consented for linkage to HES records. After excluding participants with any missing covariates, the final sample sizes differed across the exposure periods but were between 9137 and 9593 for self-reported general health and between 5524 and 5757 for hospital episodes (Fig. 2). Follow-up for hospital episodes ended at the average age of 19 years. Sample characteristics of the ‘*Infancy*’ subsamples for the two main outcomes are presented in Table 1 and show close to identical distributions after applying survey weights. Approximately 50% of the sample were female, 14% belonged to ethnic minorities, and 43% of households had highest educational levels (i.e., NVQ4-5). In the self-reported general health sample, 84% of participants were living in England, 3%, 8%, and 5% in Northern Ireland, Scotland, and Wales, respectively (Table 1). While approximately 26% of the sample had excellent general health at age 17, 7% reported to have ‘Fair or Poor’ health (Supplementary Table 2); number of hospital episodes across the different subsample are shown in Supplementary Table 3. Participants with higher number of hospital episodes reported worse general health (Supplementary Table 4). The weighted average exposure to air pollution decreased markedly during the follow up time but they remained above the 2021 WHO guideline^1^ threshold values of 5 µg m^−3^ for PM_2.5_, 15 µg m^−3^ for PM_10_, and 10 µg m^−3^ for NO_2_ (Supplementary Table 5).

**Table 1:** Weighted sample characteristics for the ‘*Infancy’* subsamples, millennium cohort study.

Variables	(A) Self-reported General health (*n* = 9593)	(B) Number of hospital episodes (*n* = 5652)
Time-invariant		
Sex, % (n)		
Female	50.2 (4878)	49.2 (2832)
Male	49.8 (4715)	50.8 (2820)
Age (years) at interview, mean ± SD	17.2 ± 0.3	17.2 ± 0.3
Ethnic group, % (n)		
White	86.7 (7817)	85.3 (4166)
Non-white	13.3 (1776)	14.7 (1486)
Number of siblings, % (n)		
None	43.2 (4099)	43.1 (2397)
1	35.5 (3319)	35.4 (1942)
2 or more	21.3 (2175)	21.5 (1313)
Partnership status, % (n)		
Single parent	15.7 (1226)	15.2 (670)
Living with partner, % (n)	84.3 (8367)	84.8 (4982)
Highest household education, % (n)		
None, overseas only	11.2 (1016)	11.1 (683)
NVQ 1–2	30.2 (2644)	31.2 (1588)
NVQ 3	15.0 (1498)	14.8 (820)
NVQ 4–5	43.7 (4435)	42.8 (2561)
Household tenure, % (n)		
Own	62.1 (6282)	61.6 (3607)
Social rent	22.5 (2037)	22.7 (1247)
Private rent	10.0 (728)	10.5 (475)
Other	5.5 (546)	5.3 (323)
Household employment, % (n)		
Both employed	41.5 (4105)	41.1 (2306)
One employed	53.1 (4947)	53.8 (3021)
None unemployed	5.4 (541)	5.1 (325)
Time-variant		
Population density, mean ± SD	42.2 ± 40.3	43.8 ± 41.0
Area-level income deprivation, % (n)		
1—Most deprived	30.7 (3545)	31.4 (2245)
2	23.7 (2265)	24.7 (1383)
3	22.9 (1921)	22.9 (1077)
4—Least deprived	22.6 (1862)	21.0 (947)
Country, % (n)		
England	84.2 (6272)	100 (5652)
Northern Ireland	3.1 (906)	NA
Scotland	7.6 (1038)	NA
Wales	5.1 (1377)	NA

*NVQ* National Vocational Qualifications, *SD* Standard Deviation.

### Air pollution and self-reported general health

**Fig. 3:**
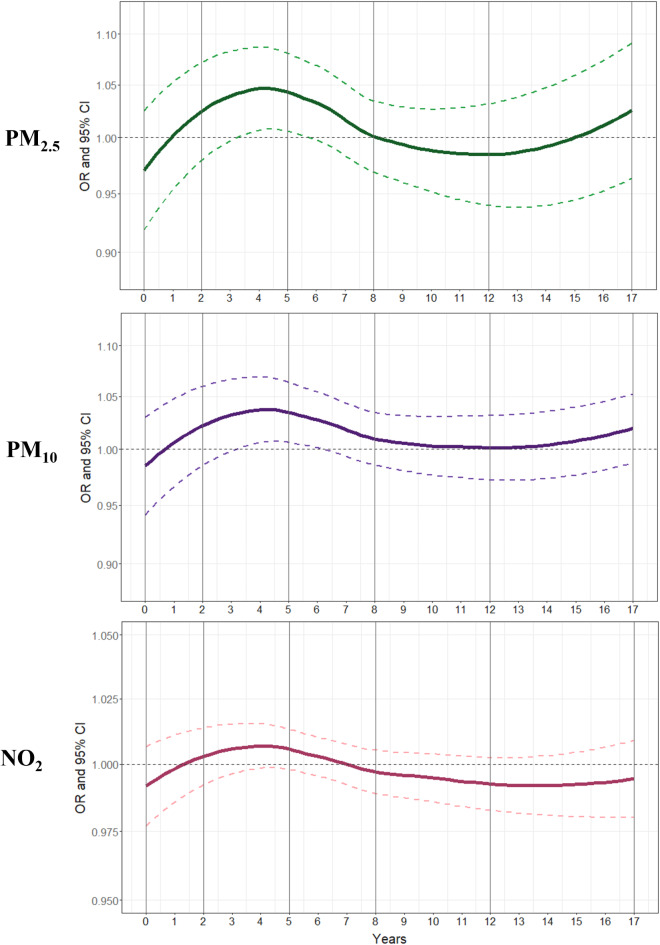
Annual PM_2.5_, PM_10_ and NO_2_ exposure from birth onwards and smoothed Odds Ratios of worse self-reported general health at age 17, Millennium Cohort Study. All models were adjusted for age, sex, ethnic groups, number of siblings, highest household education, household tenure, household employment, and partnership status; time-varying data on country, area-level income deprivation and population density were included. Complex survey weights were implemented to approximate the target population. LOESS regression was used to fit a smooth curve. ORs and their 95% CIs are smoothed and reported per 1 µg m^−3^ increment; vertical grey lines separate developmental periods.

Higher air pollution exposure was found among those reporting worse general health (Supplementary Fig. 2). After adjusting for age and sex (i.e. Model 1), PM_2.5_ and PM_10_ exposures across the whole childhood and adolescence, as well as accumulated air pollution was significantly associated with worse self-reported general health at age 17 (Table 2). Further adjusting for individual-level sociodemographic confounders (i.e. Model 2) attenuated the associations but exposure in early and middle childhood, as well as the accumulated exposure for PM_10_ remained significant. After taking into consideration area-level time-variant confounders (i.e. Model 3), we found that higher early and middle childhood exposures were significantly associated with worse general health: PM_2.5_ (OR = 1.06 per 1-µg m^−3^; 95% CI: 1.01, 1.11), PM_10_ (OR = 1.05 per 1-µg m^−3^, 95% CI: 1.01, 1.09) and NO_2_ (OR = 1.01 per 1-µg m^−3^, 95% CI: 1.01, 1.02) in early childhood, PM_2.5_ (OR = 1.04 per 1-µg m^−3^; 95% CI: 1.01, 1.07) and PM_10_ (OR = 1.03 per 1-µg m^−3^, 95% CI: 1.00, 1.06) in middle childhood (Table 2). Findings expressed in IQR change can be found in Supplementary Table 6. Post-hoc analyses showed that for PM_2.5_ and PM_10_ exposure between the ages of 3.5 and 5.5 there was a potential sensitive period, while for NO_2_ associations were not significant (Fig. 3).

**Table 2:** Air pollution exposure from birth onwards, self-reported general health and number of hospital episodes, millennium cohort study.

(A) Self-reported general health^**a**^	Model 1			Model 2			Model 3		
	OR	95% CI	*p*	OR	95% CI	*p*	OR	95% CI	*p*
PM_2.5_									
Infancy	1.02	0.99, 1.06	0.180	1.00	0.96, 1.04	0.971	0.98	0.92, 1.03	0.380
Early childhood	**1.08**	**1.05**,** 1.11**	**< 0.001**	**1.06**	**1.03**,** 1.10**	**< 0.001**	**1.06**	**1.01**,** 1.11**	**0.015**
Middle childhood	**1.05**	**1.03**,** 1.07**	**< 0.001**	**1.03**	**1.01**,** 1.06**	**0.005**	**1.04**	**1.00**,** 1.07**	**0.037**
Late childhood	**1.03**	**1.01**,** 1.06**	**0.011**	1.01	0.99, 1.04	0.321	0.99	0.95, 1.03	0.510
Adolescence	**1.05**	**1.01**,** 1.08**	**0.009**	1.02	0.98, 1.06	0.464	0.99	0.94, 1.05	0.844
Accumulation	**1.04**	**1.01**,** 1.08**	**0.011**	1.02	0.98, 1.06	0.397	0.99	0.94, 1.04	0.646
PM_10_									
Infancy	1.02	1.00, 1.05	0.101	1.00	0.98, 1.03	0.735	0.98	0.94, 1.03	0.500
Early childhood	**1.05**	**1.03**,** 1.07**	**< 0.001**	**1.04**	**1.02**,** 1.06**	**< 0.001**	**1.05**	**1.01**,** 1.09**	**0.009**
Middle childhood	**1.03**	**1.02**,** 1.05**	**< 0.001**	**1.02**	**1.01**,** 1.04**	**0.001**	**1.03**	**1.00**,** 1.06**	**0.021**
Late childhood	**1.03**	**1.01**,** 1.05**	**0.001**	1.02	1.00, 1.04	0.103	1.00	0.98, 1.03	0.854
Adolescence	**1.04**	**1.02**,** 1.06**	**< 0.001**	1.02	1.00, 1.04	0.123	1.01	0.97, 1.04	0.714
Accumulation	**1.04**	**1.02**,** 1.06**	**< 0.001**	**1.02**	**1.00**,** 1.05**	**0.045**	1.01	0.98, 1.04	0.583
NO_2_									
Infancy	1.00	1.00, 1.01	0.181	1.00	0.99, 1.01	0.576	0.99	0.98, 1.01	0.294
Early childhood	**1.02**	**1.01**,** 1.02**	**< 0.001**	**1.01**	**1.00**,** 1.02**	**0.001**	**1.01**	**1.00**,** 1.02**	**0.036**
Middle childhood	**1.01**	**1.01**,** 1.02**	**< 0.001**	1.01	1.00, 1.01	0.060	1.00	0.99, 1.01	0.579
Late childhood	**1.01**	**1.00**,** 1.02**	**0.001**	1.00	0.99, 1.01	0.551	0.99	0.99, 1.00	0.276
Adolescence	**1.01**	**1.00**,** 1.02**	**0.008**	1.00	0.99, 1.01	0.917	0.99	0.98, 1.00	0.130
Accumulation	**1.01**	**1.00**,** 1.02**	**0.024**	1.00	0.99, 1.01	0.924	0.99	0.98, 1.00	0.172

(B) Number of hospital episodes^**b**^	Model 1			Model 2			Model 3		
	IRR	95% CI	*p*	IRR	95% CI	*p*	IRR	95% CI	*p*
PM_2.5_									
Infancy	1.00	0.96, 1.04	0.869	0.99	0.95, 1.04	0.692	0.98	0.94, 1.02	0.308
Early childhood	1.02	0.95, 1.11	0.558	1.01	0.93, 1.10	0.778	0.99	0.92, 1.07	0.797
Middle childhood	1.01	0.93, 1.10	0.787	1.01	0.91, 1.12	0.835	0.97	0.90, 1.05	0.472
Late childhood	0.96	0.89, 1.03	0.233	0.95	0.88, 1.02	0.163	0.94	0.87, 1.02	0.118
Adolescence	1.20	0.97, 1.47	0.087	**1.22**	**1.01**,** 1.49**	**0.041**	**1.28**	**1.02**,** 1.61**	**0.033**
Accumulation	1.02	0.96, 1.09	0.452	1.02	0.96, 1.09	0.489	1.00	0.95, 1.06	0.966
PM_10_									
Infancy	0.99	0.96, 1.03	0.770	0.99	0.95, 1.03	0.625	0.98	0.94, 1.02	0.370
Early childhood	0.98	0.94, 1.03	0.450	0.98	0.93, 1.03	0.439	0.96	0.92, 1.00	0.073
Middle childhood	0.98	0.93, 1.04	0.537	0.98	0.92, 1.05	0.608	0.95	0.90, 1.00	0.061
Late childhood	0.96	0.90, 1.02	0.151	0.95	0.90, 1.01	0.094	0.95	0.90, 1.00	0.072
Adolescence	1.07	0.97, 1.17	0.184	1.08	0.98, 1.19	0.110	1.12	0.98, 1.27	0.087
Accumulation	0.99	0.95, 1.02	0.486	0.99	0.95, 1.03	0.566	0.97	0.93, 1.00	0.083
NO_2_									
Infancy	1.00	0.99, 1.02	0.718	1.00	0.99, 1.01	0.944	1.00	0.98, 1.02	0.912
Early childhood	1.00	0.99, 1.01	0.817	1.00	0.98, 1.02	0.954	0.99	0.98, 1.01	0.271
Middle childhood	1.00	0.99, 1.02	0.542	1.01	0.98, 1.03	0.600	0.99	0.98, 1.01	0.350
Late childhood	1.00	0.99, 1.02	0.552	1.00	0.98, 1.01	0.722	0.99	0.98, 1.01	0.406
Adolescence	1.03	0.99, 1.07	0.148	1.04	1.00, 1.08	0.058	**1.05**	**1.00**,** 1.11**	**0.046**
Accumulation	1.00	0.99, 1.01	0.800	1.00	0.99, 1.02	0.842	0.99	0.98, 1.01	0.302

Analyses were conducted using ordinal logistic regression for self-reported general health and quasi-Poisson regression for hospital episodes; complex survey weights were implemented to approximate the target population. Odds Ratios (OR), Incidence Rate Ratios (IRR) and their 95% confidence intervals (CI) are reported per 1 µg m^−3^ increment. Sample sizes are presented in the flowchart (see Fig. 2).

Model 1: adjusted for age and sex.

Model 2: Model 1 + ethnic groups, number of siblings, highest household education, household tenure, household employment, and partnership status (time-invariant).

Model 3: Model 2 + country (for self-reported general health only), area-level income deprivation and population density (time-varying).

^a^United Kingdom.

^b^England.

Significant values are in bold.

### Air pollution and number of hospital episodes

Higher PM_2.5_ and NO_2_ exposure in adolescence was associated with higher number of hospital episodes from age 18 onwards, but only in the adjusted models. 1-µg m^−3^ higher of PM_2.5_ increased the likelihood of having a hospital episode with 28% (95% CI: 1.02, 1.61), while 1-µg m^−3^ higher of NO_2_ with 5% (95% CI: 1.00, 1.11) in the fully adjusted models (Table 2). However, as linked HES data were available until 31.03.2020, the follow up time for young adulthood hospital episodes was approximately 1.8 years with an average of 0.2 episodes reported during this period (Supplementary Table 3). There were no significant associations between PM_10_ exposures and number of hospital episodes (Table 2).

### Exploring environmental health inequalities

Weighted average air pollution exposures differed between socioeconomic groups: children from non-White ethnic backgrounds, from lower parental education and those living in deprived areas had consistently higher PM_2.5_, PM_10_ and NO_2_ exposures during the first 18 years of their lives (Supplementary Fig. 3 for PM_2.5_; Supplementary Fig. 4 for PM_10_, Supplementary Fig. 5 for NO_2_). Differences were particularly striking between ethnic groups: non-White children were exposed to 15% higher PM_2.5_, 13% higher PM_10_ and a staggering 51% higher NO_2_ concentrations during early life, in comparison to White children (Supplementary Table 7). Investigating effect modification by these variables, however, suggested only minimal differences in terms of effect magnitude. There was no significant interaction with sex (Supplementary Table 8) or ethnic groups (Supplementary Table 9) after adjusting for false discovery rate.

As there was some effect modification by highest household education (Supplementary Table 10) and area deprivation (Supplementary Table 11), we plotted stratified models to visualise differences. Higher PM_2.5_, PM_10_, and NO_2_ exposure in infancy among children born to lower educated families significantly lowered the odds of worse self-reported health (Supplementary Fig. 6A). Associations between air pollution (PM_2.5_, PM_10_, NO_2_) in infancy and early childhood and self-reported general health were strongest among those living in middle deprived areas (Q3) suggesting an inverted U-shaped curve of association (Supplementary Fig. 6B–E). Finally, there were negative associations between PM_2.5_ and PM_10_ exposure in late childhood and number of hospital episodes among those living in the most deprived neighbourhoods (Q1) (Supplementary Fig. 6F–H).

### Sensitivity analyses

*S1*: Extracting area-weighted average air pollution exposure in 100-m (Supplementary Table 12) or 500-m buffers (Supplementary Table 13) around residential postcodes produced the same findings; only associations with NO_2_ attenuated using 500-m buffers. *S2*: After adjusting for maternal health (i.e. BMI before pregnancy, smoking, self-reported general health) associations minimally changed with early childhood PM_2.5_ and self-reported general health association being attenuated, while higher early childhood and accumulated PM_10_ exposure being significantly associated with lower total number of hospital episodes (Supplementary Table 14). *S3*: After adjusting for road traffic noise, main associations for PM_2.5_ and PM_10_ and self-reported general health remained significant, but attenuated for NO_2_; also, accumulated PM_10_ exposure was associated with lower total number of hospital episodes (Supplementary Table 15). *S4*: Imputing missing individual and area-level variables reinforced the main findings (Supplementary Table 16). *S5*: Analysing the life-course association between air pollution exposure and self-reported general health using multinomial regression confirmed a sensitive period around early and middle childhood: in comparison to ‘Excellent’, higher air pollution in early and middle childhood increased the likelihood of reporting ‘Good’ or ‘Fair or Poor’ general health. Moreover, the associations increased stepwise by higher PM_2.5_, PM_10_ and NO_2_ concentrations for these age groups (Supplementary Fig. 7). *S6*: In comparison to ‘Excellent’, quantile-based g-computation of exposure mixtures confirmed that air pollution during early, middle—and for fair and poor health—in late childhood was associated with lower self-reported health at age 17.

## Discussion

Higher PM_2.5_, PM_10_ and—in a smaller extend—NO_2_ exposures in early and middle childhood were associated with higher risk of reporting worse general health in a nationally representative sample of 17-year olds from the UK. Associations between air pollution and number of hospital episodes were limited, but suggested a positive link between air pollution exposure during adolescence and subsequent hospital episodes. Importantly, we found that children from disadvantaged ethnic groups, households, and neighbourhoods had higher air pollution exposures during the life; however, apart from a few exemptions, effect sizes were comparable between advantaged and disadvantaged children suggesting limited effect modification, and only for parental education and neighbourhood deprivation.

By extracting annual exposure for the first 18 years of life, we were able to identify a potential sensitive period around early and middle childhood (likely between age 3 and 6 years) for self-reported general health outcomes. Cohort studies suggest that childhood exposure may associate with adolescence or adulthood health conditions, including respiratory^37^, cardiovascular^17^, cognitive^38^ and mental health outcomes^9^. Previous findings from the Millennium Cohort Study also suggested that air pollution exposure (including PM_2.5_, PM_10_, NO_2_) at age 5 were associated with lower scores in Naming Vocabulary, a cognitive test^39^, with a French cohort study reinforcing a similar sensitive window for cognition^18^. Preschool age is considered as a sensitive period for brain development related to cognitive and behavioural changes^40^ and for lung growth^41^. Contrary to the literature^42^, air pollution exposure during infancy was not associated with self-reported general health in the total population; it was significant only after we stratified models by individual and area-level socioeconomic status. It is plausible that prenatal exposures, which we did not capture, might have a more prominent link to on general health than exposure during the first year; however, this needs to be explored in future studies. Focussing on specific conditions, where development during the first 1000 days of life is crucial^43,44^, instead of general health, might lead to different sensitive windows.

There was some evidence for adolescence air pollution exposure (PM_2.5_, NO_2_) being significantly associated with higher number of hospital episodes during young adulthood. However, given that the follow-up time was much shorter in young adulthood than in other exposure periods, we cannot ascertain whether findings were due to short versus long-term effects of air pollution on hospitalizations, or related to time of exposure during the life course; thus, this finding should be interpreted carefully. Although short and long-term air pollution exposure has been associated with hospital admission in the general population and among children, including for respiratory conditions^45^, there is evidence suggesting different associations between short and long-term air pollution exposures and health^46^. In this study, we only used the number of hospital episodes as an outcome to provide an objective marker of general health. Despite self-reported and administrative data derived general health being associated with each other, we did not find any indication for early or middle childhood air pollution exposure being linked to higher hospital episodes. Future studies interested in the link between air pollution and health in MCS and linked hospital episode data should focus on using ICD codes and detailed information on self-reported health conditions.

Unlike several longitudinal studies in the literature, our study is based on a nationally representative sample including various individual and area-level confounders. There were significant inequalities in exposure to air pollution across social and, especially, ethnic groups, which remained consistent from birth to young adulthood. The environmental injustice literature has long documented the disproportional effects of air pollution exposure on marginalised communities^20,47,48^. For disadvantaged children, the impact of environmental hazards might be particularly detrimental: air pollution can lead to developing chronic conditions in childhood which may harm their health across the whole life course^11,47^. A recent whole population study from the Netherlands found consistent differences between ethnic Dutch and minority ethnic populations^21^. PM_2.5_ exposures were 1.1–6.6%, PM_10_ exposures 0.7–5.5%, NO_2_ exposures 2.8–22.8% larger in minority populations, which are much smaller differences in comparison to those presented in the current investigation. The study also showed that air pollution exposures were higher at younger ages (< 25 years old)^21^. A US-based study confirmed ethnic group-based inequalities in air pollution exposure, adding that low-income non-White children are particularly affected by environmental inequalities^49^. Finally, a large UK-based general population sample showed higher levels of air pollution exposure among ethnic minorities and immigrants in comparison to White and UK-born individuals, which modified associations with general health^22^.

In contrast to the literature^18,22^, we found limited evidence on different effect sizes across males or females, or across social and ethnic groups. First, findings suggested stronger air pollution and self-reported general health associations in neighbourhoods which were neither advantaged nor disadvantaged (i.e., inverted U-shaped association). Second, higher air pollution during infancy was associated with better health among children growing up in household where parents had no education. We also found that among children residing in deprived neighbourhoods in late childhood, air pollution was linked to smaller number of hospital admissions. These contradictory findings require further investigation in the specific types of health conditions or that geographic accessibility might play a role.

### Strengths and limitations

This study benefitted from a large and nationally representative cohort of children followed up regularly from birth to age 17. Residential addresses were asked up to seven times (plus birth addresses) from each household, enabling us to derive air pollution exposure in each year of participants’ lives. MCS has rich individual-level information about participants and their families; further linkage to time-varying area-level exposures enabled to more accurately adjusting for confounding. A further strength of our study is the linkage of very high-resolution air pollution maps to residential postcodes, which are able to reduce exposure misclassification in comparison to earlier representative longitudinal studies using larger geographies^22,39^. However, there are several limitations to consider. First, there is still some risk of misclassifying air pollution exposure, as we did not have the exact property addresses geocoded. Using buffers around postcode centroids may lower the distance error and thus exposure misclassification, however, with increasing sizes of buffers localised effects of air pollutants become harder to capture. This might be less problematic in remote areas where air pollution is likely low and more homogeneous, but could introduce problems in high density populated areas. In addition, we do not have information about daily mobility patterns which are key to define the real area of exposure. Second, there are significant differences in how area-level confounders were produced between the four UK nations and across time. For example, small areas have slightly different populations (i.e., smallest in Scotland), and indices of multiple deprivation were produced in different years. While we adjusted models for country of residence, we cannot rule out different measurements biasing findings. Third, although 85% of the sample consented to health data linkage in Sweep7 and the weighted sample distribution for the two outcomes were almost identical, there is evidence suggesting differences between consenting and not-consenting survey participants^50^, which is not included in the survey weights. Fourth, general health was derived from participants’ self-report, which is prone to reporting bias. Fifth, despite identifying a range of relevant confounders, residual confounding cannot be ruled out due to imperfect selection of covariates (e.g. policy impact) or measurement error. Last, analyses in this paper do not examine the causal relationship between air pollution and general health; future studies should implement quasi-experimental designs or instrumental variable approaches to strengthen evidence base.

## Conclusions

Using a large and nationally representative sample, this study demonstrated how PM_2.5_, PM_10_ and NO_2_ exposure during early and middle childhood associates with general health in late adolescence. Findings extend on previous literature suggesting a sensitive window and exploring environmental inequalities across social and ethnic groups. Future research should utilise representative birth cohorts linked with environmental and health administrative data to understand how air pollution is associated with specific health conditions and identify disease-specific sensitive windows. Confirming sensitive windows across cohorts from different generations and regions, and exploring causality using quasi-experimental studies should be prioritized. As air pollution exposure in childhood is linked to health and well-being in later life, policies reducing concentrations below WHO guideline limits may have benefits across the whole life course.

## Electronic supplementary material

Below is the link to the electronic supplementary material.

Supplementary Material 1

## Data Availability

All MCS datasets are available via the UK Data Service (https://beta.ukdataservice.ac.uk/datacatalogue/series/series?id=2000031); air pollution maps were produced by the EXPANSE project and are available upon request (https://expanseproject.eu/toolbox/exposome-maps/). Researchers interested in linking environmental data to MCS postcodes can apply for data access via CLS Data Access Committee (https://cls.ucl.ac.uk/data-access-training/data-access/accessing-data-directly-from-cls/); completed application should be submitted to clsdata@ucl.ac.uk. Further queries can be directed to corresponding author.
