# Supplementary material for: The relationship between early life course air pollution exposure and general health in adolescence in the United Kingdom
Source: Sci Rep. 2025 May 14;15:10983. doi: 10.1038/s41598-025-94107-w (PMC12078791; doi:10.1038/s41598-025-94107-w)

Early life-course exposure to air pollution, environmental inequality and general health at age 17: a national cohort study from the United Kingdom

Gergő Baranyi, Katie Harron, Youchen Shen, Kees de Hoogh, Emla Fitzsimons

**Supplementary Material**

**Supplementary Figure 1:** Exposure-specific correlation maps between across ages (A) and across developmental periods (B).

**Supplementary Table 1**: Source of linked environmental data.

**Supplementary Table 2**: Weighted percentage of self-reported general health status across samples, Millennium Cohort Study.

**Supplementary Table 3**: Weighted average number of hospital episodes across samples, Millennium Cohort Study.

**Supplementary Table 4**: Number of hospital episodes by self-reported general health categories, Millennium Cohort Study.

**Supplementary Table 5:** Weighted average and standard deviation of air pollution exposure in specific developmental periods (µg m^-3^)

**Supplementary Figure 2**: Weighted average PM_2.5_, PM_10_, and NO_2_ exposure from birth onwards by levels of self-reported general health at age 17.

**Supplementary Figure 3**: Weighted average PM_2.5_ exposure across (A) sex, (B) ethnicity, (C) highest household qualifications and (D) area-level income deprivation at Sweep 1, Millennium Cohort Study.

**Supplementary Figure 4**: Weighted average PM_10_ exposure across (A) sex, (B) ethnicity, (C) highest household qualifications and (D) area-level income deprivation at Sweep 1, Millennium Cohort Study.

**Supplementary Figure 5**: Weighted average NO_2_ exposure across (A) sex, (B) ethnicity, (C) highest household qualifications and (D) area-level income deprivation at Sweep 1, Millennium Cohort Study.

**Supplementary Table 6**: Air pollution exposure from birth onwards, self-reported general health and number of hospital episodes with association expressed as interquartile range increase in the fully-adjusted models, Millennium Cohort Study.

**Supplementary Table 7**: Accumulated air pollution exposure across sociodemographic groups, Millennium Cohort Study.

**Supplementary Table 8**: Interaction between air pollution exposure and sex (reference female) on self-reported general health and number of hospital episodes in the fully-adjusted models, Millennium Cohort Study.

**Supplementary Table 9**: Interaction between air pollution exposure and ethnicity (reference white) on self-reported general health and number of hospital episodes in the fully-adjusted models, Millennium Cohort Study.

**Supplementary Table 10**: Interaction between air pollution exposure and highest household education (reference none or overseas only) on self-reported general health and number of hospital episodes in the fully-adjusted models, Millennium Cohort Study.

**Supplementary Table 11**: Interaction between air pollution exposure and area-level income deprivation (reference Q1 – Most deprived) on self-reported general health and number of hospital episodes in the fully-adjusted model, Millennium Cohort Study.

**Supplementary Figure 6**: Stratified analyses between air pollution, self-reported general health and number of hospital episodes, across (A) educational and (B-H) deprivation groups in the fully-adjusted models, Millennium Cohort Study.

**Supplementary Table 12**: Air pollution exposure using 100m buffers, self-reported general health and number of hospital episodes, Millennium Cohort Study.

**Supplementary Table 13**: Air pollution exposure using 500m buffers, self-reported general health and number of hospital episodes, Millennium Cohort Study.

**Supplementary Table 14**: Air pollution exposure from birth onwards, self-reported general health and number of hospital episodes after adjusting for maternal health, Millennium Cohort Study.

**Supplementary Table 15**: Air pollution exposure from birth onwards, self-reported general health and number of hospital episodes after adjusting for road traffic noise, Millennium Cohort Study.

**Supplementary Table 16**: Air pollution exposure from birth onwards, self-reported general health and number of hospital episodes after multiple imputation in the fully-adjusted models, Millennium Cohort Study.

**Supplementary Figure 7**: Multinomial regression showing the odds of reporting ‘very good’, ‘good’ and ‘fair or poor’ general health at age 17 by higher PM_2.5_, PM_10_ and NO_2_ exposure from birth onwards, Millennium Cohort Study.

**Supplementary Figure 8**: Quantile g-computation for multinomial outcomes showing the odds of reporting ‘very good’, ‘good’ and ‘fair or poor’ general health at age 17 by higher air pollution exposure (mixture of PM_2.5_, PM_10_ and NO_2_) from birth onwards, Millennium Cohort Study.

**Supplementary Figure 1:** Exposure-specific correlation maps between across ages (A) and across developmental periods (B). Figures are based on all individuals participating in sweep 7.

(A)


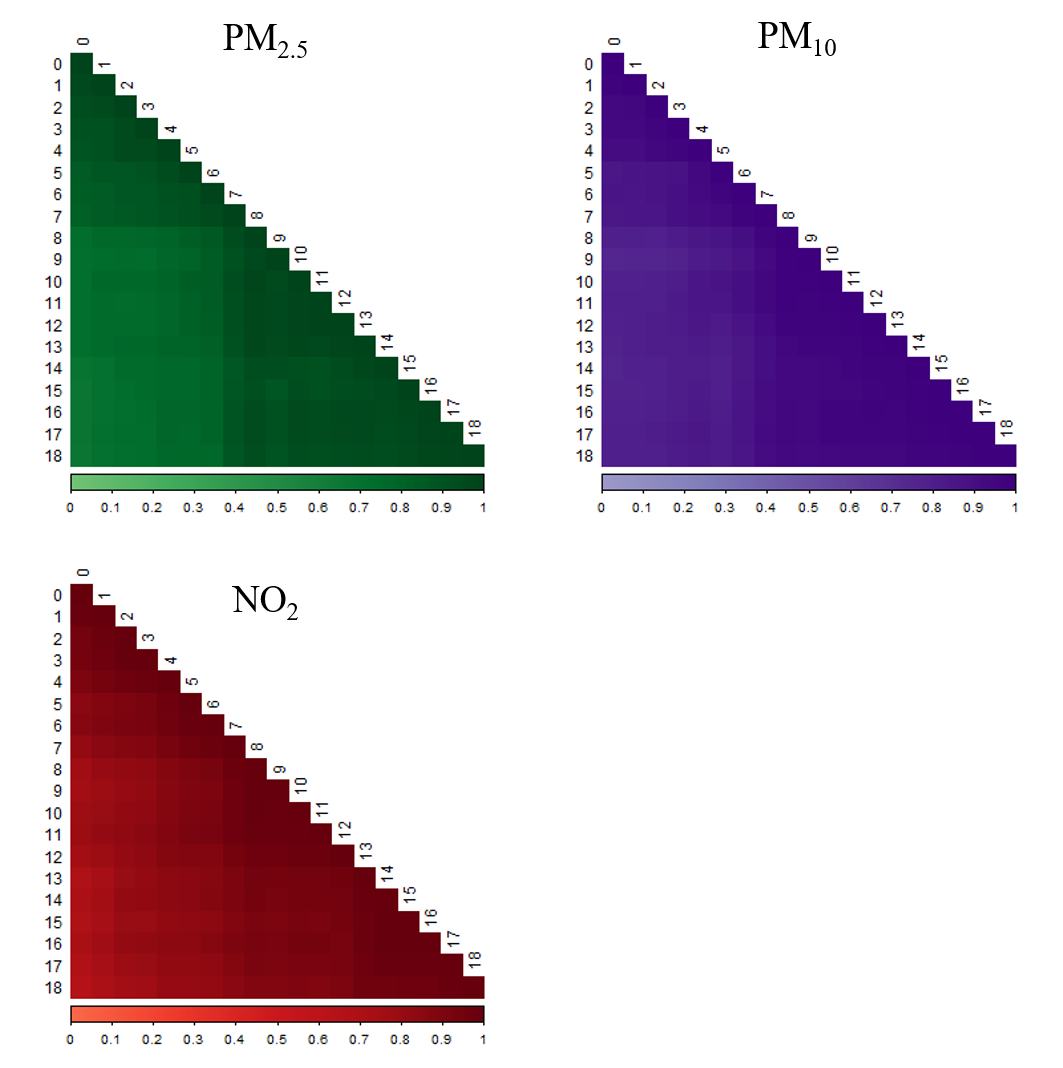


(B)


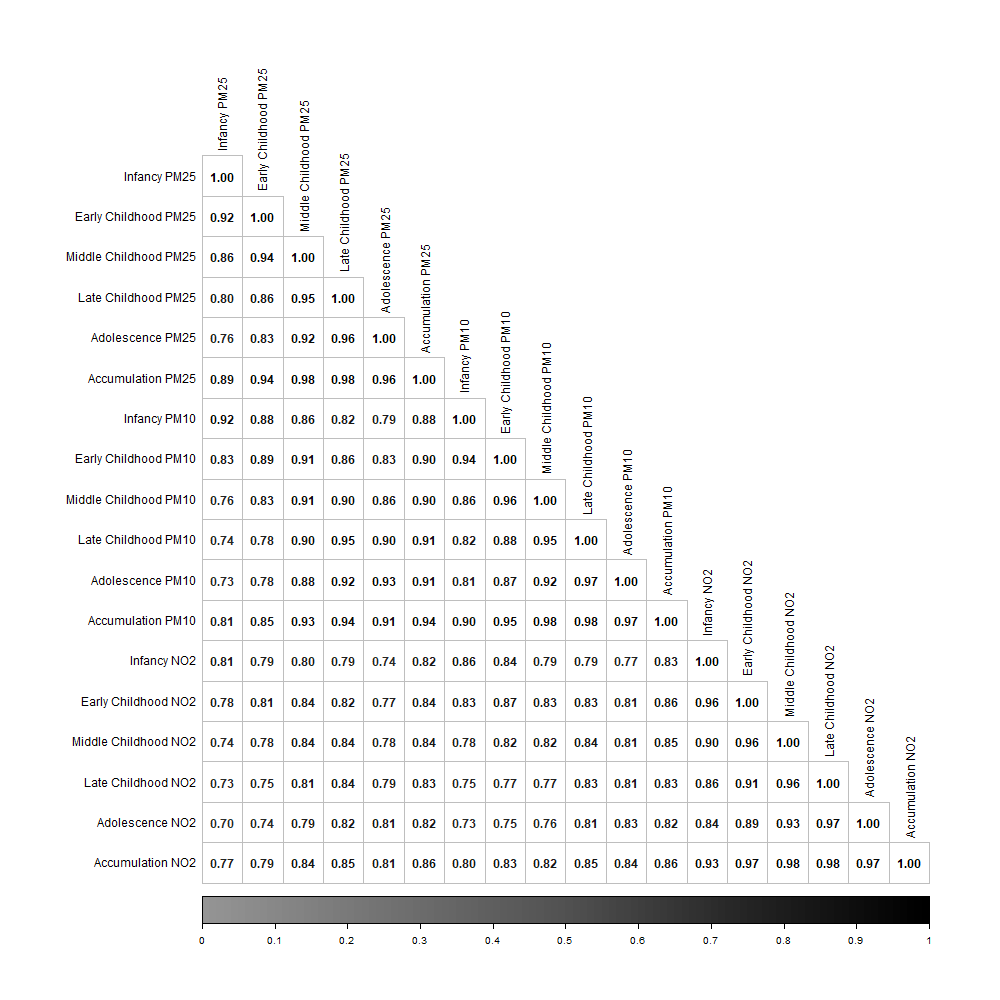


**Supplementary Table 1**: Source of linked environmental data.

| **Area** | **Year** | **Sweep** | **Data resolution** | **Source** |
| --- | --- | --- | --- | --- |
| **﻿ Population density** | | | | |
| England | 2001 | 1, 2, 3, 4, 5 | Lower layer Super Output Areas | <https://www.nomisweb.co.uk/datasets/uv002> |
| England | 2011 | 6, 7 | Lower layer Super Output Areas | <https://www.nomisweb.co.uk/census/2011/qs102uk> |
| Northern Ireland | 2001 | 1, 2, 3, 4, 5 | Super Output Areas | <https://www.nisra.gov.uk/statistics/2001-and-earlier-censuses/2001-census> |
| Northern Ireland | 2011 | 6, 7 | Super Output Areas | <https://www.nisra.gov.uk/statistics/census/2011-census> |
| Scotland | 2001 | 1, 2, 3, 4, 5 | Data zone | <https://www.opendata.nhs.scot/nl/dataset/population-estimates/resource/bf086aee-130d-4487-b854-808db0e29dc4> |
| Scotland | 2011 | 6, 7 | Data zone | <https://www.nomisweb.co.uk/census/2011/qs102uk> |
| Wales | 2001 | 1, 2, 3, 4, 5 | Lower layer Super Output Areas | <https://www.nomisweb.co.uk/datasets/uv002> |
| Wales | 2001 | 6, 7 | Lower layer Super Output Areas | <https://www.nomisweb.co.uk/census/2011/qs102uk> |
| **Road traffic noise exposure (Lden)** | | | | |
| England | 2012 | 1, 2, 3, 4, 5, 6, 7 | Major roads (>3m vehicle annually); Roads in agglomerations (>100.000 people) | <https://environment.data.gov.uk/dataset/2ec0abc3-ec1d-459c-aa6e-573672e94da7> |
| Northern Ireland | 2012 | 1, 2, 3, 4, 5, 6, 7 | Major roads (>3m vehicle annually); Roads in agglomerations (>100.000 people) | <https://www.daera-ni.gov.uk/services/noise-maps> |
| Scotland | 2012 | 1, 2, 3, 4, 5, 6, 7 | Major roads (>3m vehicle annually); Roads in agglomerations (>100.000 people) | <https://noise.environment.gov.scot/noisemap> |
| Wales | 2012 | 1, 2, 3, 4, 5, 6, 7 | Major roads (>3m vehicle annually); Roads in agglomerations (>100.000 people) | <https://datamap.gov.wales/layergroups/inspire-wg:EnvironmentalNoiseMapping> |
| **Income deprivation ranks** | | | | |
| England | 2004 | 1, 2 | Lower layer Super Output Areas | <https://www.data.gov.uk/dataset/59599787-bd50-4500-a409-fc586260dbbd/index-of-multiple-deprivation-2004> |
| England | 2007 | 3, 4 | Lower layer Super Output Areas | <https://www.data.gov.uk/dataset/bdc1e1a5-aaf3-4f5a-9988-82a11e341eb8/index-of-multiple-deprivation-imd-2007> |
| England | 2010 | 5 | Lower layer Super Output Areas | <https://www.gov.uk/government/statistics/english-indices-of-deprivation-2010> |
| England | 2015 | 6 | Lower layer Super Output Areas | <https://www.gov.uk/government/statistics/english-indices-of-deprivation-2015> |
| England | 2019 | 7 | Lower layer Super Output Areas | <https://www.gov.uk/government/statistics/english-indices-of-deprivation-2019> |
| Northern Ireland | 2005 | 1, 2, 3 | Super Output Areas | <https://www.nisra.gov.uk/statistics/deprivation/northern-ireland-multiple-deprivation-measure-2005-nimdm2005> |
| Northern Ireland | 2010 | 4, 5 | Super Output Areas | <https://www.nisra.gov.uk/statistics/deprivation/northern-ireland-multiple-deprivation-measure-2010-nimdm2010> |
| Northern Ireland | 2017 | 6, 7 | Super Output Areas | <https://www.nisra.gov.uk/statistics/deprivation/northern-ireland-multiple-deprivation-measure-2017-nimdm2017> |
| Scotland | 2004 | 1, 2 | Data zone | <https://www.data.gov.uk/dataset/535abdb8-b3d6-4802-ab9a-7646c417158c/scottish-index-of-multiple-deprivation-simd-2004#:~:text=The%20Scottish%20Index%20of%20Multiple,the%20Scottish%20Government's%20first%20edition> |
| Scotland | 2006 | 3 | Data zone | <https://www.data.gov.uk/dataset/06daf95e-593f-447d-918b-7b72afcb0b76/scottish-index-of-multiple-deprivation-simd-2006> |
| Scotland | 2009 | 4 | Data zone | <https://www.data.gov.uk/dataset/0782efa6-554a-4896-aa54-d1ec23067532/scottish-index-of-multiple-deprivation-simd-2009> |
| Scotland | 2012 | 5 | Data zone | <https://www.data.gov.uk/dataset/d9a46acf-ae72-4dc4-b17c-af76222fd6f6/scottish-index-of-multiple-deprivation-simd-2012> |
| Scotland | 2016 | 6 | Data zone | <https://www.data.gov.uk/dataset/a448dd2a-9197-4ea0-8357-c2c9b3c29591/scottish-index-of-multiple-deprivation-simd-2016> |
| Scotland | 2020 | 7 | Data zone | <https://www.data.gov.uk/dataset/1102bf85-ed49-440a-b211-da87e8d752eb/scottish-index-of-multiple-deprivation-simd-2020> |
| Wales | 2005 | 1, 2 | Lower layer Super Output Areas | <https://statswales.gov.wales/Catalogue/Community-Safety-and-Social-Inclusion/Welsh-Index-of-Multiple-Deprivation/Archive/WIMD-2005> |
| Wales | 2008 | 3 | Lower layer Super Output Areas | <https://statswales.gov.wales/Catalogue/Community-Safety-and-Social-Inclusion/Welsh-Index-of-Multiple-Deprivation/Archive/WIMD-2008> |
| Wales | 2011 | 4 | Lower layer Super Output Areas | <https://statswales.gov.wales/Catalogue/Community-Safety-and-Social-Inclusion/Welsh-Index-of-Multiple-Deprivation/Archive/WIMD-2011> |
| Wales | 2014 | 5, 6 | Lower layer Super Output Areas | <https://statswales.gov.wales/Catalogue/Community-Safety-and-Social-Inclusion/Welsh-Index-of-Multiple-Deprivation/Archive/WIMD-2014> |
| Wales | 2019 | 7 | Lower layer Super Output Areas | <https://statswales.gov.wales/Catalogue/Community-Safety-and-Social-Inclusion/Welsh-Index-of-Multiple-Deprivation/WIMD-2019> |
| **Air pollution (PM_2.5_, PM_10_)** | | | | |
| United Kingdom | 2000-2019 | All | 25m × 25m grids | <https://expanseproject.eu/> |

**Supplementary Table 2:** Weighted percentage of self-reported general health status across samples, Millennium Cohort Study.

|  | Total sample^a^  (n=9971) | Subsamples (by exposure periods)^b^ | | | | | |
| --- | --- | --- | --- | --- | --- | --- | --- |
|  |  | Infancy  (n=9593) | Early Childhood (n=9137) | Middle Childhood (n=9171) | Late Childhood (n=9422) | Adolescence (n=9216) | Accumulation (n=9216) |
| Excellent, % (n) | 26.3 (2874) | 26.3 (2782) | 27.6 (2670) | 27.8 (2661) | 27.8 (2741) | 28.7 (2698) | 28.7 (2698) |
| Very good, % (n) | 41.8 (3945) | 42.5 (3805) | 39.8 (3598) | 40.8 (3648) | 40.6 (3731) | 40.3 (3659) | 40.3 (3659) |
| Good, % (n) | 24.9 (2448) | 24.4 (2333) | 25.4 (2234) | 24.7 (2233) | 24.4 (2284) | 24.1 (2213) | 24.1 (2213) |
| Fair or poor, % (n) | 7.0 (704) | 6.8 (673) | 7.2 (635) | 6.7 (629) | 7.1 (666) | 7.0 (646) | 7.0 (646) |

*Note*: Percentages might not sum up to 100 due to rounding errors.

^a^ No missing for the outcome

^b^ Complete cases

**Supplementary Table 3:** Weighted average number of hospital episodes across samples, Millennium Cohort Study.

| Hospital episodes until 31.03.2020  (median age of 19) | Total sample^a^  (n=6104) | Subsamples (by exposure periods)^b^ | | | | | |
| --- | --- | --- | --- | --- | --- | --- | --- |
|  |  | Infancy  (n=5652) | Early Childhood (n=5534) | Middle Childhood (n=5524) | Late Childhood (n=5757) | Adolescence (n=5641) | Accumulation (n=5641) |
| Total episodes, mean ± SD | 2.4 ± 5.8 | - | - | - | - | - | 2.2 ± 4.7 |
| Early Childhood (2y) onwards, mean ± SD | 1.8 ± 5.2 | 1.8 ± 5.3 | - | - | - | - | - |
| Middle Childhood (5y) onwards, mean ± SD | 1.5 ± 4.6 | - | 1.3 ± 4.2 | - | - | - | - |
| Late Childhood (8y) onwards, mean ± SD | 1.2 ± 4.2 | - | - | 1.1 ± 4.0 | - | - | - |
| Adolescence (12y) onwards, mean ± SD | 0.8 ± 3.4 | - | - | - | 0.8 ± 2.8 | - | - |
| Young Adulthood (18y) onwards, mean ± SD | 0.2 ± 1.1 | - | - | - | - | 0.2 ± 1.1 | - |

^a^ No missing for the outcome

^b^ Complete cases

**Supplementary Table 4**: Number of hospital episodes by self-reported general health categories, Millennium Cohort Study.

|  | Excellent | Very Good | Good | Fair or Poor |
| --- | --- | --- | --- | --- |
| Total episodes, mean ± SD | 1.8 ± 3.2 | 1.8 ± 4.2 | 3.8 ± 8.6 | 3.9 ± 7.5 |
| Early Childhood (2y) onwards, mean ± SD | 1.2 ± 3.0 | 1.3 ± 4.0 | 2.9 ± 7.5 | 3.2 ± 7.1 |
| Middle Childhood (5y) onwards, mean ± SD | 0.9 ± 1.9 | 1.0 ± 3.8 | 2.4 ± 6.4 | 2.8 ± 6.9 |
| Late Childhood (8y) onwards, mean ± SD | 0.7 ± 1.5 | 0.8 ± 3.7 | 2.0 ± 5.7 | 2.4 ± 6.6 |
| Adolescence (12y) onwards, mean ± SD | 0.5 ± 1.2 | 0.6 ± 3.4 | 1.2 ± 3.8 | 1.9 ± 5.8 |
| Young Adulthood (18y) onwards, mean ± SD | 0.1 ± 0.4 | 0.1 ± 0.8 | 0.4 ± 1.8 | 0.4 ± 1.3 |

Sample is based on no missing for hospital episode outcomes (n=6104).

**Supplementary Table 5:** Weighted average and standard deviation of air pollution exposure in specific developmental periods (µg m^-3^), Millennium Cohort Study

|  | Self-reported general health |  | Number of hospital episodes |
| --- | --- | --- | --- |
|  | Mean ± SD |  | Mean ± SD |
| **PM_2.5_** | | | |
| Infancy | 13.0 ± 2.3 |  | 13.5 ± 1.9 |
| Early Childhood | 12.8 ± 2.0 |  | 13.3 ± 1.5 |
| Middle Childhood | 12.1 ± 2.2 |  | 12.7 ± 1.6 |
| Late Childhood | 11.0 ± 2.2 |  | 11.5 ± 1.7 |
| Adolescence | 9.6 ± 1.7 |  | 10.1 ± 1.3 |
| Accumulation | 11.3 ± 1.9 |  | 11.8 ± 1.5 |
| **PM_10_** | | | |
| Infancy | 23.6 ± 2.8 |  | 24.3 ± 2.2 |
| Early Childhood | 23.2 ± 2.9 |  | 24.0 ± 2.2 |
| Middle Childhood | 21.5 ± 3.2 |  | 22.4 ± 2.4 |
| Late Childhood | 18.1 ± 3.2 |  | 18.8 ± 2.7 |
| Adolescence | 16.4 ± 2.6 |  | 17.0 ± 2.1 |
| Accumulation | 19.7 ± 2.8 |  | 20.3 ± 2.2 |
| **NO_2_** |  |  |  |
| Infancy | 27.5 ± 9.8 |  | 29.1 ± 9.2 |
| Early Childhood | 25.2 ± 9.4 |  | 26.6 ± 8.8 |
| Middle Childhood | 23.2 ± 9.2 |  | 24.7 ± 8.7 |
| Late Childhood | 22.0 ± 8.5 |  | 23.2 ± 8.0 |
| Adolescence | 19.2 ± 7.5 |  | 20.4 ± 7.2 |
| Accumulation | 22.6 ± 8.3 |  | 23.9 ± 7.9 |

Sample sizes are presented in the flowchart (see Figure 2). Abbreviations: SD=Standard Deviation.

**Supplementary Figure 2**: Weighted average PM_2.5_, PM_10_, and NO_2_ exposure from birth onwards by levels of self-reported general health at age 17. Dashed lines indicate 2021 WHO threshold values. Sample sizes are presented in the flowchart (see Figure 2).

PM_2.5_
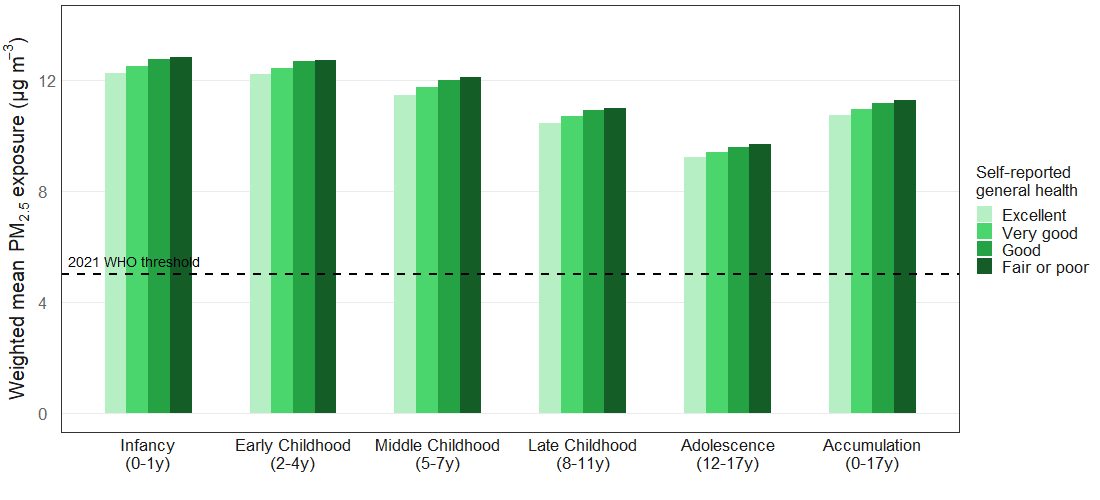


PM_10_
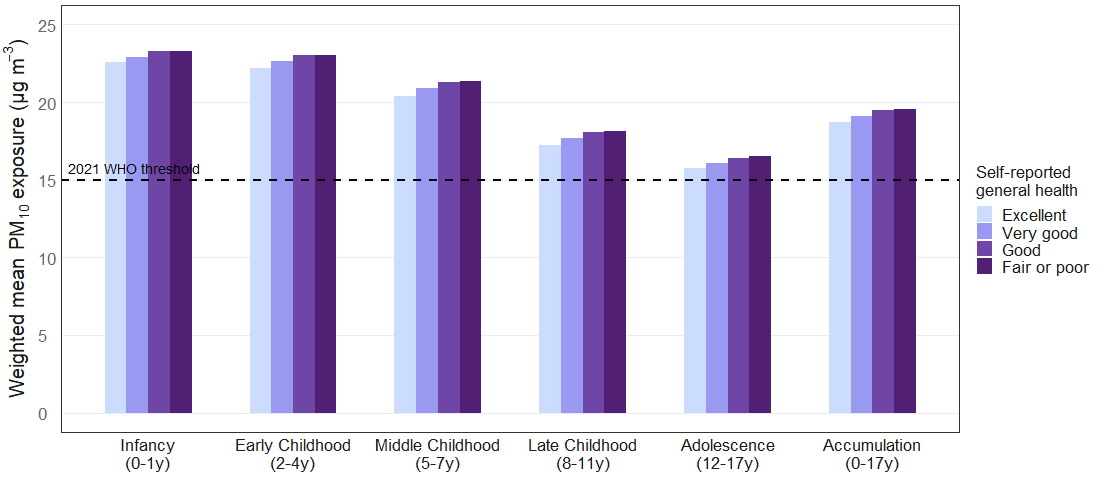


NO_2_
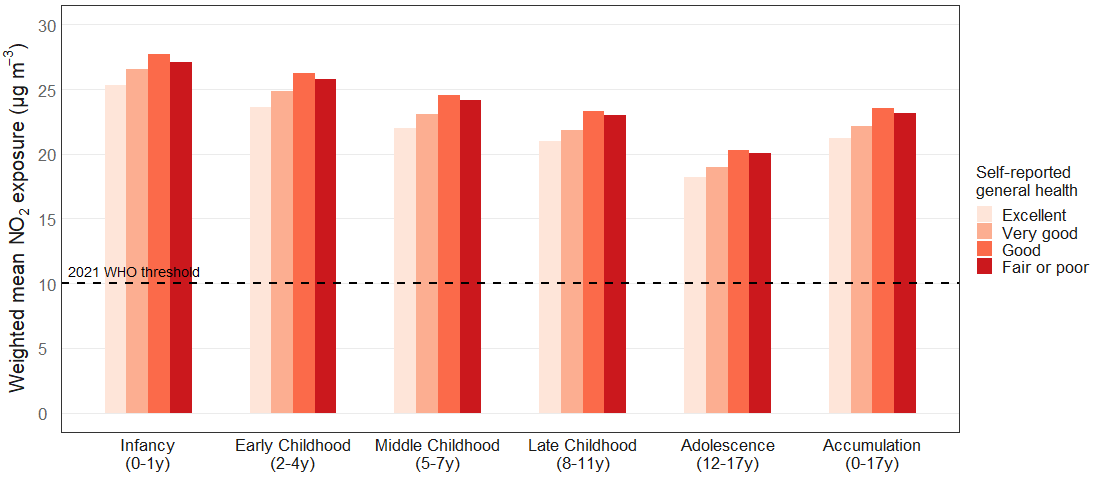


**Supplementary Table 6**: Air pollution exposure from birth onwards, self-reported general health and number of hospital episodes with association expressed as interquartile range increase in the fully-adjusted models, Millennium Cohort Study.

|  | **PM_2.5_** | | |  | **PM_10_** | | |  |  | **NO_2_** |  |
| --- | --- | --- | --- | --- | --- | --- | --- | --- | --- | --- | --- |
| Self-reported general health | OR | 95% CI | *p* |  | OR | 95% CI | *p* |  | OR | 95% CI | *p* |
| Infancy | 0.91 | 0.73, 1.13 | 0.380 |  | 0.93 | 0.74, 1.16 | 0.500 |  | 0.90 | 0.73, 1.10 | 0.294 |
| Early Childhood | **1.21** | **1.04, 1.41** | **0.015** |  | **1.29** | **1.06, 1.56** | **0.009** |  | **1.15** | **1.01, 1.32** | **0.036** |
| Middle Childhood | **1.14** | **1.01, 1.30** | **0.037** |  | **1.16** | **1.02, 1.32** | **0.021** |  | 1.03 | 0.94, 1.12 | 0.579 |
| Late Childhood | 0.95 | 0.82, 1.10 | 0.510 |  | 1.01 | 0.88, 1.17 | 0.854 |  | 0.94 | 0.84, 1.05 | 0.276 |
| Adolescence | 0.98 | 0.84, 1.16 | 0.844 |  | 1.02 | 0.90, 1.17 | 0.713 |  | 0.90 | 0.79, 1.03 | 0.130 |
| Accumulation | 0.96 | 0.79, 1.16 | 0.646 |  | 1.05 | 0.89, 1.23 | 0.583 |  | 0.90 | 0.77, 1.05 | 0.172 |
| Number of hospital episodes | IRR | 95% CI | *p* |  | IRR | 95% CI | *p* |  | IRR | 95% CI | *p* |
| Infancy | 0.92 | 0.78, 1.08 | 0.308 |  | 0.92 | 0.76, 1.11 | 0.370 |  | 0.99 | 0.79, 1.24 | 0.912 |
| Early Childhood | 0.97 | 0.75, 1.24 | 0.797 |  | 0.80 | 0.63, 1.02 | 0.073 |  | 0.9 | 0.74, 1.09 | 0.271 |
| Middle Childhood | 0.90 | 0.67, 1.21 | 0.472 |  | 0.78 | 0.60, 1.01 | 0.061 |  | 0.91 | 0.74, 1.11 | 0.350 |
| Late Childhood | 0.79 | 0.59, 1.06 | 0.118 |  | 0.76 | 0.57, 1.02 | 0.072 |  | 0.93 | 0.77, 1.11 | 0.406 |
| Adolescence | **2.02** | **1.06, 3.83** | **0.033** |  | 1.58 | 0.94, 2.66 | 0.087 |  | **1.74** | **1.01, 3.00** | **0.046** |
| Accumulation | 1.00 | 0.83, 1.22 | 0.966 |  | 0.85 | 0.71, 1.02 | 0.083 |  | 0.93 | 0.80, 1.07 | 0.302 |

Analyses were conducted using ordinal logistic regression for self-reported general health and quasi-Poisson regression for hospital episodes; complex survey weights were implemented to approximate the target population. Odds Ratios (OR), Incidence Rate Ratios (IRR) and their 95% confidence intervals (CI) are reported. Sample sizes are presented in the flowchart (see Figure 2).

All models were adjusted for age, sex, ethnic groups, number of siblings, highest household education, household tenure, household employment, and partnership status. Time-varying data on country (for self-reported general health only), area-level income deprivation and population density were included from the respective exposure period.

**Supplementary Figure 3:** Weighted average PM_2.5_ exposure across (A) sex, (B) ethnicity, (C) highest household qualifications and (D) area-level income deprivation at Sweep 1, Millennium Cohort Study. Dashed lines indicate 2021 WHO threshold values. Figures are based on all individuals participating in sweep 7.


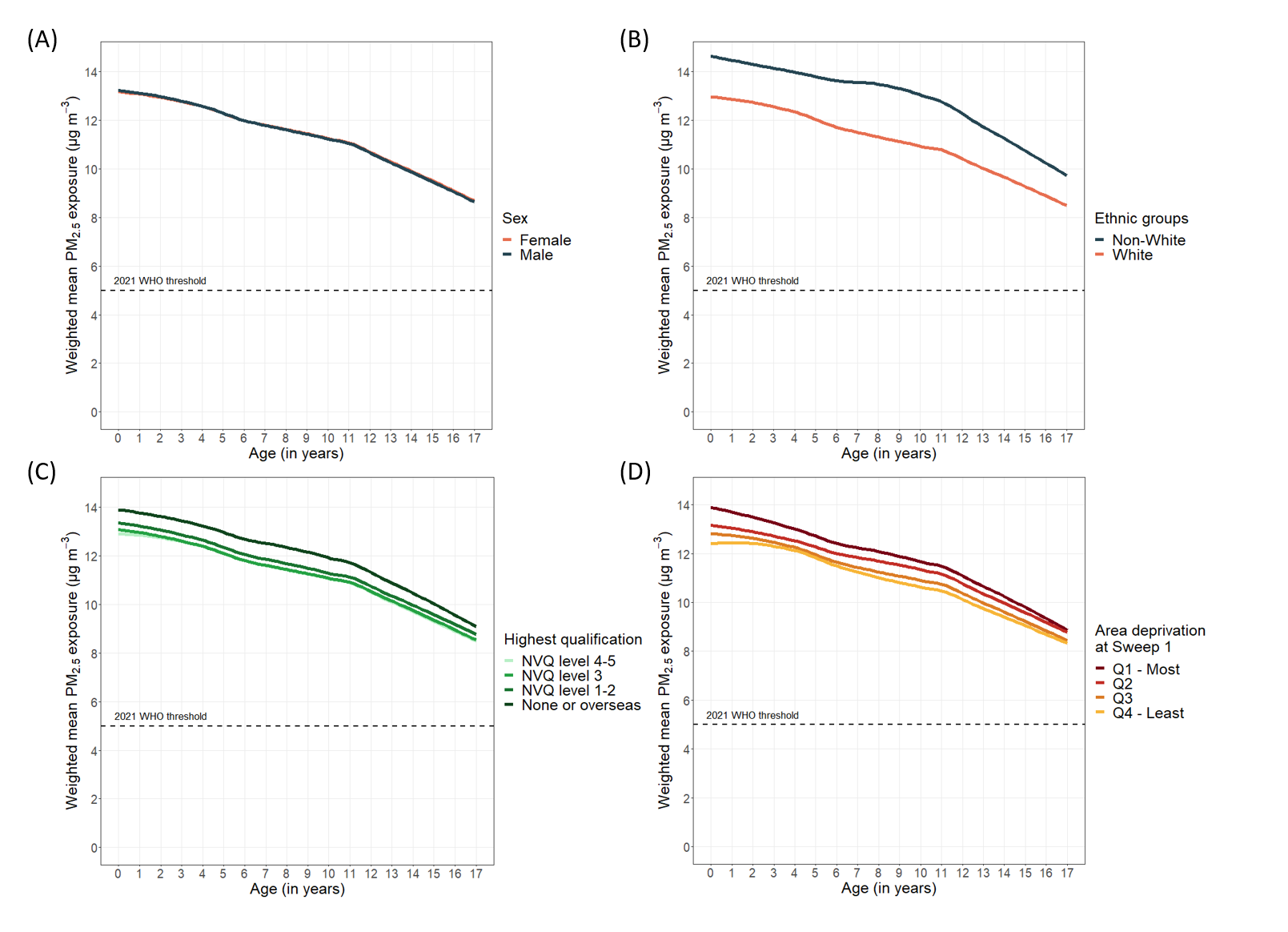


**Supplementary Figure 4:** Weighted average PM_10_ exposure across (A) sex, (B) ethnicity, (C) highest household qualifications and (D) area-level income deprivation at Sweep 1, Millennium Cohort Study. Dashed lines indicate 2021 WHO threshold values. Figures are based on all individuals participating in sweep 7.


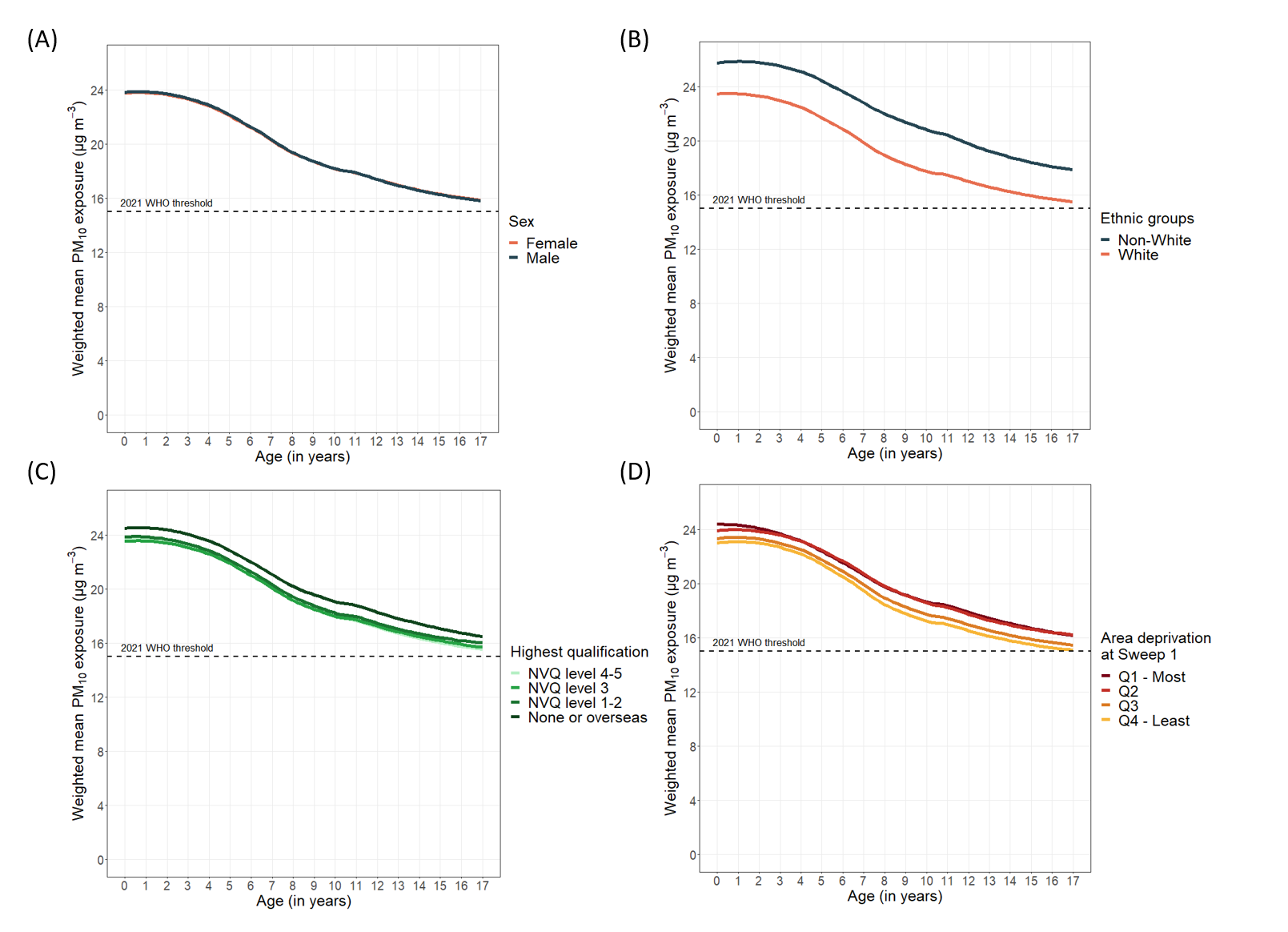


**Supplementary Figure 5:** Weighted average NO_2_ exposure across (A) sex, (B) ethnicity, (C) highest household qualifications and (D) area-level income deprivation at Sweep 1, Millennium Cohort Study. Dashed lines indicate 2021 WHO threshold values. Figures are based on all individuals participating in sweep 7.


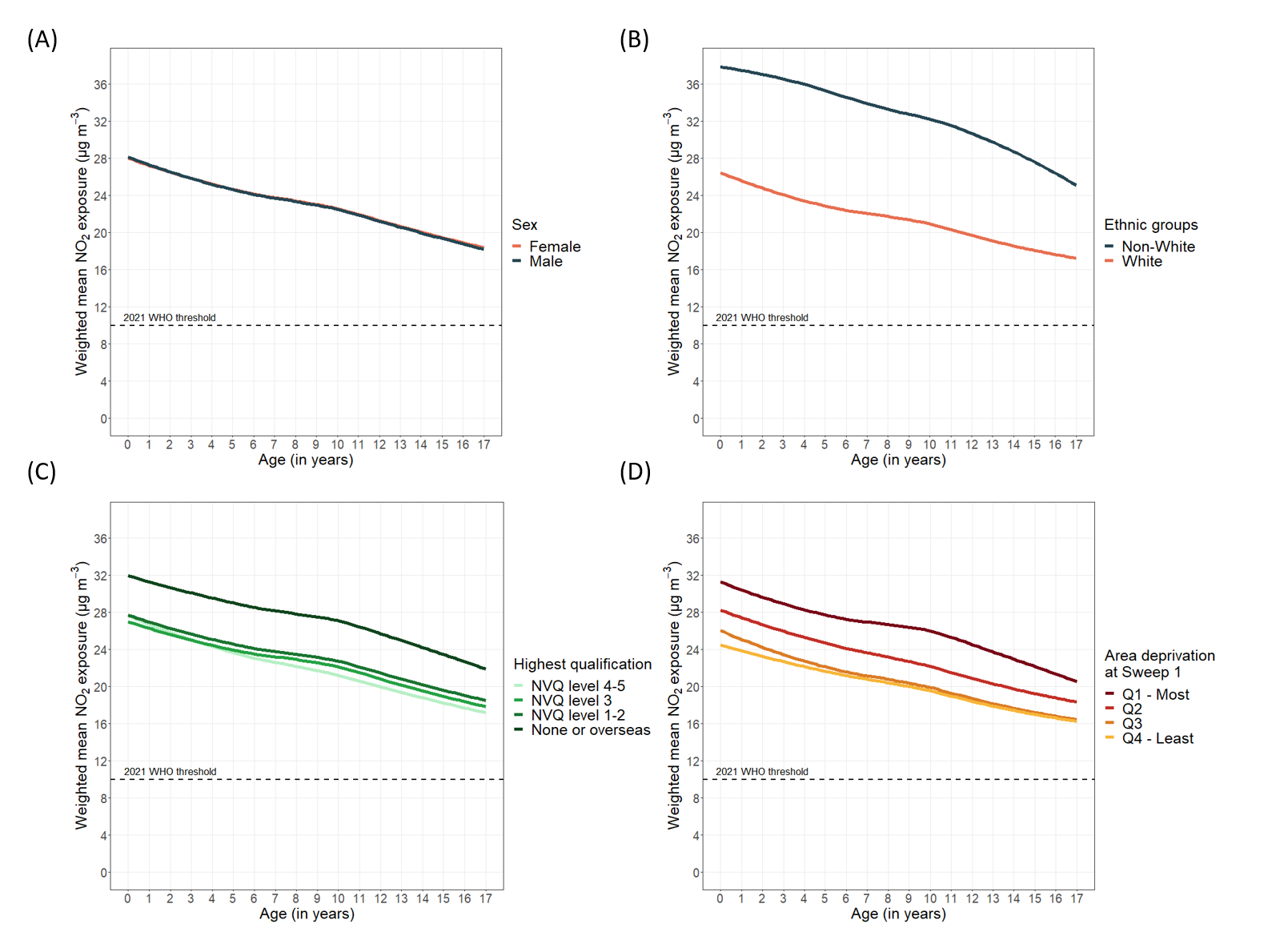


**Supplementary Table 7:** Accumulated air pollution exposure across sociodemographic groups, Millennium Cohort Study.

| Groups | PM_2.5_ exposure | |  | PM_10_ exposure | |  | NO_2_ exposure | |
| --- | --- | --- | --- | --- | --- | --- | --- | --- |
|  | Mean | 95% CI |  | Mean | 95% CI |  | Mean | 95% CI |
| Sex |  |  |  |  |  |  |  |  |
| Male | 11.3 | 11.1, 11.5 |  | 19.7 | 19.4, 20.0 |  | 22.7 | 21.7, 23.7 |
| Female | 11.2 | 11.1, 11.4 |  | 19.6 | 19.3, 19.9 |  | 22.4 | 21.5, 23.4 |
| Ethnic groups |  |  |  |  |  |  |  |  |
| White | 11.1 | 10.9, 11.3 |  | 19.3 | 19.1, 19.6 |  | 21.2 | 20.4, 22.0 |
| Non-White | 12.8 | 12.6, 13.0 |  | 21.9 | 21.5, 22.4 |  | 32.1 | 30.6, 33.7 |
| Highest household education | | | | | | | | |
| None, overseas only | 12.1 | 11.9, 12.4 |  | 20.7 | 20.4, 21.1 |  | 27.8 | 26.3, 29.2 |
| NVQ 1-2 | 11.4 | 11.2, 11.6 |  | 19.7 | 19.4, 20.0 |  | 22.8 | 21.8, 23.8 |
| NVQ 3 | 11.1 | 10.9, 11.4 |  | 19.5 | 19.2, 19.8 |  | 21.9 | 20.9, 22.9 |
| NVQ 4-5 | 11.1 | 10.9, 11.3 |  | 19.5 | 19.1, 19.8 |  | 21.7 | 20.6, 22.9 |
| Area-level income deprivation | | | | | | | | |
| 1 - Most deprived | 11.8 | 11.5, 12.0 |  | 20.2 | 19.9, 20.6 |  | 26.2 | 24.8, 27.6 |
| 2 | 11.4 | 11.2, 11.7 |  | 20.0 | 19.6, 20.4 |  | 22.7 | 21.4, 24.1 |
| 3 | 11.1 | 10.8, 11.5 |  | 19.4 | 18.9, 19.8 |  | 21.0 | 19.3, 22.7 |
| 4 - Least deprived | 10.7 | 10.4, 10.9 |  | 18.8 | 18.4, 19.2 |  | 19.7 | 18.7, 20.7 |

Total sample size is n=9216.

**Supplementary Table 8**: Interaction between air pollution exposure and sex (reference female) on self-reported general health and number of hospital episodes in the fully-adjusted models, Millennium Cohort Study.

|  | **PM_2.5_** | | |  | **PM_10_** | | |  | **NO_2_** | | |
| --- | --- | --- | --- | --- | --- | --- | --- | --- | --- | --- | --- |
| Self-reported general health | OR | 95% CI | *p_FDR_* |  | OR | 95% CI | *p_FDR_* |  | OR | 95% CI | *p_FDR_* |
| Infancy | 1.00 | 0.95, 1.06 | 0.918 |  | 1.02 | 0.98, 1.07 | 0.378 |  | 1.00 | 0.99, 1.02 | 0.832 |
| Early Childhood | 1.01 | 0.96, 1.05 | 0.918 |  | 1.00 | 0.97, 1.03 | 0.964 |  | 1.00 | 0.99, 1.01 | 0.832 |
| Middle Childhood | 0.98 | 0.94, 1.01 | 0.486 |  | 0.98 | 0.96, 1.01 | 0.378 |  | 1.00 | 0.99, 1.00 | 0.552 |
| Late Childhood | 0.97 | 0.93, 1.02 | 0.486 |  | 0.98 | 0.95, 1.01 | 0.378 |  | 0.99 | 0.98, 1.01 | 0.552 |
| Adolescence | 0.96 | 0.91, 1.02 | 0.486 |  | 0.97 | 0.94, 1.01 | 0.378 |  | 0.99 | 0.97, 1.00 | 0.528 |
| Accumulation | 0.97 | 0.92, 1.03 | 0.486 |  | 0.98 | 0.95, 1.02 | 0.378 |  | 0.99 | 0.98, 1.00 | 0.528 |
| Number of hospital episodes | IRR | 95% CI | *p_FDR_* |  | IRR | 95% CI | *p_FDR_* |  | IRR | 95% CI | *p_FDR_* |
| Infancy | 0.99 | 0.92, 1.06 | 0.958 |  | 1.01 | 0.93, 1.10 | 0.908 |  | 1.00 | 0.98, 1.02 | 0.976 |
| Early Childhood | 1.00 | 0.86, 1.18 | 0.958 |  | 1.00 | 0.90, 1.11 | 0.991 |  | 1.01 | 0.98, 1.04 | 0.972 |
| Middle Childhood | 1.04 | 0.86, 1.26 | 0.958 |  | 1.03 | 0.91, 1.16 | 0.908 |  | 1.02 | 0.98, 1.06 | 0.837 |
| Late Childhood | 1.05 | 0.91, 1.2 | 0.958 |  | 1.04 | 0.95, 1.14 | 0.908 |  | 1.00 | 0.98, 1.02 | 0.976 |
| Adolescence | 0.98 | 0.78, 1.24 | 0.958 |  | 1.03 | 0.88, 1.21 | 0.908 |  | 1.01 | 0.98, 1.04 | 0.972 |
| Accumulation | 1.01 | 0.91, 1.12 | 0.958 |  | 1.02 | 0.95, 1.09 | 0.908 |  | 1.01 | 0.99, 1.03 | 0.837 |

Analyses were conducted using ordinal logistic regression for self-reported general health and quasi-Poisson regression for hospital episodes; complex survey weights were implemented to approximate the target population. Odds Ratios (OR), Incidence Rate Ratios (IRR) and their 95% confidence intervals (CI) are reported per 1 µg m^-3^ increment. Sample sizes are presented in the flowchart (see Figure 2).

All models were adjusted for age, sex, ethnic groups, number of siblings, highest household education, household tenure, household employment, and partnership status. Time-varying data on country (for self-reported general health only), area-level income deprivation and population density were included from the respective exposure period.

**Supplementary Table 9**: Interaction between air pollution exposure and ethnicity (reference white) on self-reported general health and number of hospital episodes in the fully-adjusted models, Millennium Cohort Study.

|  | **PM_2.5_** | | |  | **PM_10_** | | |  | **NO_2_** | | |
| --- | --- | --- | --- | --- | --- | --- | --- | --- | --- | --- | --- |
| Self-reported general health | OR | 95% CI | *p_FDR_* |  | OR | 95% CI | *p_FDR_* |  | OR | 95% CI | *p_FDR_* |
| Infancy | 0.98 | 0.88, 1.1 | 0.994 |  | 0.97 | 0.9, 1.05 | 0.993 |  | 1.00 | 0.98, 1.01 | 0.920 |
| Early Childhood | 1.01 | 0.89, 1.15 | 0.994 |  | 0.99 | 0.92, 1.06 | 0.993 |  | 0.99 | 0.97, 1.00 | 0.732 |
| Middle Childhood | 1.06 | 0.96, 1.17 | 0.994 |  | 1.01 | 0.95, 1.07 | 0.993 |  | 1.00 | 0.98, 1.01 | 0.920 |
| Late Childhood | 1.00 | 0.92, 1.09 | 0.994 |  | 1.01 | 0.96, 1.06 | 0.993 |  | 0.99 | 0.98, 1.01 | 0.920 |
| Adolescence | 1.02 | 0.91, 1.14 | 0.994 |  | 1.00 | 0.94, 1.07 | 0.993 |  | 1.00 | 0.98, 1.02 | 0.920 |
| Accumulation | 1.04 | 0.92, 1.16 | 0.994 |  | 1.01 | 0.94, 1.07 | 0.993 |  | 1.00 | 0.98, 1.02 | 0.920 |
| Number of hospital episodes | IRR | 95% CI | *p_FDR_* |  | IRR | 95% CI | *p_FDR_* |  | IRR | 95% CI | *p_FDR_* |
| Infancy | 1.14 | 1.01, 1.29 | 0.168 |  | 0.95 | 0.83, 1.08 | 0.414 |  | 0.97 | 0.95, 1.00 | 0.202 |
| Early Childhood | 0.93 | 0.76, 1.14 | 0.463 |  | 0.87 | 0.73, 1.04 | 0.246 |  | 0.97 | 0.94, 1.00 | 0.202 |
| Middle Childhood | 0.81 | 0.57, 1.16 | 0.353 |  | 0.80 | 0.64, 1.00 | 0.246 |  | 0.97 | 0.93, 1.01 | 0.202 |
| Late Childhood | 0.86 | 0.64, 1.14 | 0.353 |  | 0.88 | 0.73, 1.08 | 0.339 |  | 0.97 | 0.93, 1.01 | 0.219 |
| Adolescence | 0.71 | 0.50, 1.01 | 0.168 |  | 0.90 | 0.70, 1.16 | 0.414 |  | 0.98 | 0.92, 1.04 | 0.416 |
| Accumulation | 0.87 | 0.72, 1.05 | 0.310 |  | 0.89 | 0.78, 1.03 | 0.246 |  | 0.98 | 0.96, 1.01 | 0.274 |

Analyses were conducted using ordinal logistic regression for self-reported general health and quasi-Poisson regression for hospital episodes; complex survey weights were implemented to approximate the target population. Odds Ratios (OR), Incidence Rate Ratios (IRR) and their 95% confidence intervals (CI) are reported per 1 µg m^-3^ increment. Sample sizes are presented in the flowchart (see Figure 2).

All models were adjusted for age, sex, ethnic groups, number of siblings, highest household education, household tenure, household employment, and partnership status. Time-varying data on country (for self-reported general health only), area-level income deprivation and population density were included from the respective exposure period.

**Supplementary Table 10**: Interaction between air pollution exposure and highest household education (reference none or overseas only) on self-reported general health and number of hospital episodes in the fully-adjusted models, Millennium Cohort Study.

|  | **PM_2.5_** | | |  | **PM_10_** | | |  | **NO_2_** | | |
| --- | --- | --- | --- | --- | --- | --- | --- | --- | --- | --- | --- |
| Self-reported general health | OR | 95% CI | *p_FDR_* |  | OR | 95% CI | *p_FDR_* |  | OR | 95% CI | *p_FDR_* |
| Infancy |  |  |  |  |  |  |  |  |  |  |  |
| NVQ 1-2 | 1.21 | 1.03, 1.43 | 0.112 |  | 1.16 | 1.00, 1.33 | 0.289 |  | 1.04 | 1.01, 1.07 | 0.083 |
| NVQ 3 | 1.21 | 1.03, 1.41 | 0.108 |  | 1.14 | 0.99, 1.31 | 0.418 |  | **1.04** | **1.01, 1.07** | **0.040** |
| NVQ 4-5 | **1.24** | **1.06, 1.44** | **0.037** |  | 1.16 | 1.02, 1.32 | 0.174 |  | **1.05** | **1.02, 1.07** | **0.008** |
| Early Childhood |  |  |  |  |  |  |  |  |  |  |  |
| NVQ 1-2 | 1.10 | 0.96, 1.25 | 0.220 |  | 1.01 | 0.92, 1.10 | 0.884 |  | 1.02 | 0.99, 1.05 | 0.564 |
| NVQ 3 | 1.06 | 0.93, 1.21 | 0.458 |  | 0.98 | 0.90, 1.07 | 0.654 |  | 1.02 | 1.00, 1.05 | 0.238 |
| NVQ 4-5 | 1.07 | 0.94, 1.22 | 0.402 |  | 1.00 | 0.92, 1.08 | 0.988 |  | 1.02 | 1.00, 1.05 | 0.158 |
| Middle Childhood |  |  |  |  |  |  |  |  |  |  |  |
| NVQ 1-2 | 1.11 | 1.01, 1.23 | 0.112 |  | 1.01 | 0.94, 1.08 | 0.884 |  | 1.01 | 0.99, 1.03 | 0.651 |
| NVQ 3 | 1.09 | 0.98, 1.22 | 0.234 |  | 1.02 | 0.95, 1.10 | 0.654 |  | 1.01 | 0.99, 1.03 | 0.814 |
| NVQ 4-5 | 1.10 | 0.99, 1.23 | 0.216 |  | 1.02 | 0.95, 1.09 | 0.725 |  | 1.01 | 0.99, 1.03 | 0.579 |
| Late Childhood |  |  |  |  |  |  |  |  |  |  |  |
| NVQ 1-2 | 1.08 | 0.96, 1.21 | 0.220 |  | 1.02 | 0.94, 1.10 | 0.884 |  | 1.01 | 0.98, 1.03 | 0.651 |
| NVQ 3 | 1.10 | 0.98, 1.24 | 0.234 |  | 1.04 | 0.97, 1.13 | 0.489 |  | 1.01 | 0.99, 1.04 | 0.488 |
| NVQ 4-5 | 1.09 | 0.98, 1.22 | 0.251 |  | 1.03 | 0.96, 1.11 | 0.582 |  | 1.01 | 0.99, 1.04 | 0.579 |
| Adolescence |  |  |  |  |  |  |  |  |  |  |  |
| NVQ 1-2 | 0.90 | 0.78, 1.04 | 0.220 |  | 0.93 | 0.85, 1.01 | 0.309 |  | 0.99 | 0.96, 1.02 | 0.651 |
| NVQ 3 | 0.94 | 0.82, 1.07 | 0.458 |  | 0.96 | 0.89, 1.04 | 0.489 |  | 1.00 | 0.97, 1.02 | 0.926 |
| NVQ 4-5 | 0.94 | 0.83, 1.06 | 0.402 |  | 0.96 | 0.90, 1.02 | 0.412 |  | 1.00 | 0.99, 1.02 | 0.630 |
| Accumulation |  |  |  |  |  |  |  |  |  |  |  |
| NVQ 1-2 | 0.95 | 0.84, 1.08 | 0.450 |  | 0.95 | 0.88, 1.03 | 0.358 |  | 0.99 | 0.97, 1.02 | 0.651 |
| NVQ 3 | 0.97 | 0.86, 1.10 | 0.666 |  | 0.97 | 0.90, 1.04 | 0.489 |  | 1.00 | 0.98, 1.02 | 0.926 |
| NVQ 4-5 | 0.98 | 0.88, 1.10 | 0.751 |  | 0.96 | 0.90, 1.02 | 0.412 |  | 1.00 | 0.99, 1.02 | 0.630 |
| Number of hospital episodes | IRR | 95% CI | *p_FDR_* |  | IRR | 95% CI | *p_FDR_* |  | IRR | 95% CI | *p_FDR_* |
| Infancy |  |  |  |  |  |  |  |  |  |  |  |
| NVQ 1-2 | 1.05 | 0.92, 1.20 | 0.668 |  | 1.03 | 0.90, 1.18 | 0.780 |  | 0.99 | 0.96, 1.03 | 0.684 |
| NVQ 3 | 1.03 | 0.87, 1.22 | 0.794 |  | 0.98 | 0.84, 1.15 | 0.824 |  | 0.98 | 0.95, 1.01 | 0.783 |
| NVQ 4-5 | 1.07 | 0.93, 1.22 | 0.679 |  | 0.99 | 0.89, 1.11 | 0.860 |  | 0.98 | 0.96, 1.01 | 0.602 |
| Early Childhood |  |  |  |  |  |  |  |  |  |  |  |
| NVQ 1-2 | 1.03 | 0.79, 1.33 | 0.895 |  | 1.11 | 0.95, 1.31 | 0.391 |  | 1.02 | 0.98, 1.07 | 0.535 |
| NVQ 3 | 0.94 | 0.72, 1.24 | 0.794 |  | 1.03 | 0.85, 1.25 | 0.824 |  | 1.01 | 0.98, 1.04 | 0.920 |
| NVQ 4-5 | 0.96 | 0.77, 1.19 | 0.837 |  | 1.06 | 0.93, 1.21 | 0.646 |  | 1.01 | 0.99, 1.04 | 0.602 |
| Middle Childhood |  |  |  |  |  |  |  |  |  |  |  |
| NVQ 1-2 | 1.21 | 0.91, 1.61 | 0.642 |  | 1.11 | 0.92, 1.33 | 0.391 |  | 1.03 | 0.97, 1.09 | 0.535 |
| NVQ 3 | 1.07 | 0.84, 1.35 | 0.794 |  | 1.02 | 0.85, 1.23 | 0.824 |  | 1.00 | 0.96, 1.04 | 0.920 |
| NVQ 4-5 | 1.12 | 0.93, 1.36 | 0.679 |  | 1.05 | 0.93, 1.19 | 0.646 |  | 1.01 | 0.98, 1.04 | 0.602 |
| Late Childhood |  |  |  |  |  |  |  |  |  |  |  |
| NVQ 1-2 | 1.15 | 0.92, 1.42 | 0.642 |  | 1.10 | 0.95, 1.27 | 0.391 |  | 0.98 | 0.95, 1.01 | 0.535 |
| NVQ 3 | 1.15 | 0.87, 1.53 | 0.794 |  | 1.12 | 0.90, 1.38 | 0.824 |  | 1.00 | 0.97, 1.04 | 0.920 |
| NVQ 4-5 | 1.22 | 1.01, 1.47 | 0.229 |  | 1.18 | 1.03, 1.36 | 0.106 |  | 1.01 | 0.98, 1.03 | 0.602 |
| Adolescence |  |  |  |  |  |  |  |  |  |  |  |
| NVQ 1-2 | 0.75 | 0.42, 1.33 | 0.642 |  | 0.98 | 0.77, 1.26 | 0.900 |  | 0.97 | 0.90, 1.05 | 0.590 |
| NVQ 3 | 0.80 | 0.40, 1.59 | 0.794 |  | 1.04 | 0.77, 1.42 | 0.824 |  | 0.98 | 0.89, 1.08 | 0.920 |
| NVQ 4-5 | 0.79 | 0.40, 1.58 | 0.768 |  | 1.08 | 0.79, 1.49 | 0.734 |  | 0.97 | 0.87, 1.07 | 0.602 |
| Accumulation |  |  |  |  |  |  |  |  |  |  |  |
| NVQ 1-2 | 1.02 | 0.79, 1.30 | 0.895 |  | 1.11 | 0.96, 1.28 | 0.391 |  | 1.02 | 0.98, 1.06 | 0.535 |
| NVQ 3 | 1.03 | 0.83, 1.27 | 0.794 |  | 1.08 | 0.97, 1.20 | 0.824 |  | 1.01 | 0.99, 1.04 | 0.783 |
| NVQ 4-5 | 1.02 | 0.83, 1.27 | 0.837 |  | 1.09 | 0.99, 1.21 | 0.277 |  | 1.01 | 0.99, 1.04 | 0.602 |

Analyses were conducted using ordinal logistic regression for self-reported general health and quasi-Poisson regression for hospital episodes; complex survey weights were implemented to approximate the target population. Odds Ratios (OR), Incidence Rate Ratios (IRR) and their 95% confidence intervals (CI) are reported per 1 µg m^-3^ increment. Sample sizes are presented in the flowchart (see Figure 2).

All models were adjusted for age, sex, ethnic groups, number of siblings, highest household education, household tenure, household employment, and partnership status. Time-varying data on country (for self-reported general health only), area-level income deprivation and population density were included from the respective exposure period.

**Supplementary Table 11**: Interaction between air pollution exposure and area-level income deprivation (reference Q1 – Most deprived) on self-reported general health and number of hospital episodes in the fully-adjusted models, Millennium Cohort Study.

|  | **PM_2.5_** | | |  | **PM_10_** | | |  | **NO_2_** | | |
| --- | --- | --- | --- | --- | --- | --- | --- | --- | --- | --- | --- |
| Self-reported general health | OR | 95% CI | *p_FDR_* |  | OR | 95% CI | *p_FDR_* |  | OR | 95% CI | *p_FDR_* |
| Infancy |  |  |  |  |  |  |  |  |  |  |  |
| Q2 | 1.05 | 0.95, 1.16 | 0.411 |  | 1.03 | 0.95, 1.12 | 0.617 |  | 1.01 | 0.99, 1.03 | 0.462 |
| Q3 | 1.11 | 1.01, 1.21 | 0.094 |  | 1.07 | 1.00, 1.15 | 0.163 |  | **1.02** | **1.01, 1.04** | **0.021** |
| Q4 – Least deprived | 1.06 | 0.93, 1.20 | 0.482 |  | 1.04 | 0.95, 1.13 | 0.443 |  | 1.02 | 0.99, 1.05 | 0.200 |
| Early Childhood |  |  |  |  |  |  |  |  |  |  |  |
| Q2 | 0.98 | 0.90, 1.08 | 0.739 |  | 0.99 | 0.93, 1.06 | 0.884 |  | 1.01 | 0.99, 1.03 | 0.489 |
| Q3 | 1.01 | 0.94, 1.09 | 0.725 |  | 1.01 | 0.96, 1.07 | 0.603 |  | **1.02** | **1.01, 1.04** | **0.021** |
| Q4 – Least deprived | 1.00 | 0.92, 1.08 | 0.955 |  | 1.01 | 0.96, 1.06 | 0.715 |  | 1.02 | 1.00, 1.03 | 0.109 |
| Middle Childhood |  |  |  |  |  |  |  |  |  |  |  |
| Q2 | 1.06 | 0.99, 1.13 | 0.230 |  | 1.03 | 0.98, 1.07 | 0.413 |  | 1.01 | 0.99, 1.02 | 0.408 |
| Q3 | 1.08 | 1.01, 1.16 | 0.094 |  | 1.05 | 1.00, 1.10 | 0.163 |  | **1.02** | **1.01, 1.04** | **0.021** |
| Q4 – Least deprived | 1.06 | 0.99, 1.13 | 0.232 |  | 1.04 | 1.00, 1.09 | 0.171 |  | 1.02 | 1.00, 1.03 | 0.109 |
| Late Childhood |  |  |  |  |  |  |  |  |  |  |  |
| Q2 | 1.05 | 0.97, 1.13 | 0.358 |  | 1.00 | 0.95, 1.06 | 0.884 |  | 1.01 | 0.99, 1.03 | 0.477 |
| Q3 | 1.06 | 0.98, 1.15 | 0.253 |  | 1.02 | 0.97, 1.08 | 0.456 |  | 1.02 | 1.00, 1.04 | 0.088 |
| Q4 – Least deprived | 1.08 | 1.01, 1.17 | 0.216 |  | 1.05 | 1.00, 1.10 | 0.171 |  | 1.02 | 1.00, 1.04 | 0.109 |
| Adolescence |  |  |  |  |  |  |  |  |  |  |  |
| Q2 | 1.12 | 1.00, 1.26 | 0.230 |  | 1.07 | 1.00, 1.13 | 0.226 |  | 1.03 | 1.00, 1.05 | 0.148 |
| Q3 | 1.07 | 0.95, 1.21 | 0.338 |  | 1.03 | 0.97, 1.10 | 0.456 |  | 1.03 | 1.00, 1.05 | 0.079 |
| Q4 – Least deprived | 1.09 | 0.97, 1.24 | 0.246 |  | 1.05 | 0.99, 1.12 | 0.248 |  | 1.03 | 1.00, 1.06 | 0.109 |
| Accumulation |  |  |  |  |  |  |  |  |  |  |  |
| Q2 | 1.09 | 0.98, 1.22 | 0.230 |  | 1.05 | 1.00, 1.10 | 0.226 |  | 1.02 | 1.00, 1.05 | 0.148 |
| Q3 | 1.08 | 0.97, 1.21 | 0.253 |  | 1.03 | 0.97, 1.09 | 0.456 |  | 1.02 | 1.00, 1.05 | 0.088 |
| Q4 – Least deprived | 1.08 | 0.97, 1.21 | 0.246 |  | 1.04 | 0.98, 1.10 | 0.262 |  | 1.03 | 1.00, 1.05 | 0.109 |
| Number of hospital episodes | IRR | 95% CI | *p_FDR_* |  | IRR | 95% CI | *p_FDR_* |  | IRR | 95% CI | *p_FDR_* |
| Infancy |  |  |  |  |  |  |  |  |  |  |  |
| Q2 | 1.09 | 0.98, 1.21 | 0.301 |  | 1.08 | 0.97, 1.2 | 0.508 |  | 1.01 | 0.99, 1.04 | 0.822 |
| Q3 | 1.06 | 0.95, 1.17 | 0.846 |  | 1.00 | 0.92, 1.08 | 0.973 |  | 0.99 | 0.97, 1.01 | 0.922 |
| Q4 – Least deprived | 0.97 | 0.85, 1.11 | 0.967 |  | 0.97 | 0.87, 1.1 | 0.907 |  | 0.97 | 0.94, 1.01 | 0.533 |
| Early Childhood |  |  |  |  |  |  |  |  |  |  |  |
| Q2 | 0.96 | 0.77, 1.19 | 0.816 |  | 1.00 | 0.85, 1.18 | 0.969 |  | 0.99 | 0.95, 1.03 | 0.822 |
| Q3 | 0.98 | 0.79, 1.21 | 0.846 |  | 0.97 | 0.83, 1.13 | 0.973 |  | 1.00 | 0.95, 1.04 | 0.922 |
| Q4 – Least deprived | 0.94 | 0.74, 1.20 | 0.967 |  | 0.97 | 0.82, 1.15 | 0.907 |  | 1.00 | 0.95, 1.04 | 0.958 |
| Middle Childhood |  |  |  |  |  |  |  |  |  |  |  |
| Q2 | 0.96 | 0.69, 1.33 | 0.816 |  | 0.97 | 0.79, 1.18 | 0.969 |  | 0.98 | 0.93, 1.04 | 0.822 |
| Q3 | 0.94 | 0.68, 1.30 | 0.846 |  | 0.99 | 0.81, 1.21 | 0.973 |  | 0.98 | 0.93, 1.04 | 0.922 |
| Q4 – Least deprived | 0.98 | 0.71, 1.34 | 0.967 |  | 0.96 | 0.78, 1.18 | 0.907 |  | 0.99 | 0.94, 1.04 | 0.958 |
| Late Childhood |  |  |  |  |  |  |  |  |  |  |  |
| Q2 | 1.22 | 1.03, 1.46 | 0.153 |  | 1.15 | 1.02, 1.29 | 0.125 |  | 1.00 | 0.97, 1.04 | 0.824 |
| Q3 | 1.14 | 0.96, 1.35 | 0.746 |  | 1.12 | 1.00, 1.26 | 0.304 |  | 1.00 | 0.97, 1.04 | 0.922 |
| Q4 – Least deprived | **1.31** | **1.11, 1.54** | **0.007** |  | **1.21** | **1.05, 1.39** | **0.045** |  | 1.03 | 1.00, 1.07 | 0.198 |
| Adolescence |  |  |  |  |  |  |  |  |  |  |  |
| Q2 | 0.83 | 0.5, 1.36 | 0.816 |  | 0.98 | 0.78, 1.23 | 0.969 |  | 0.95 | 0.88, 1.03 | 0.822 |
| Q3 | 0.87 | 0.53, 1.4 | 0.846 |  | 1.00 | 0.8, 1.26 | 0.973 |  | 0.97 | 0.90, 1.06 | 0.922 |
| Q4 – Least deprived | 0.97 | 0.55, 1.73 | 0.967 |  | 1.04 | 0.78, 1.41 | 0.907 |  | 1.00 | 0.91, 1.10 | 0.958 |
| Accumulation |  |  |  |  |  |  |  |  |  |  |  |
| Q2 | 0.98 | 0.84, 1.15 | 0.816 |  | 0.97 | 0.86, 1.09 | 0.969 |  | 0.99 | 0.96, 1.02 | 0.822 |
| Q3 | 0.97 | 0.82, 1.14 | 0.846 |  | 0.97 | 0.86, 1.09 | 0.973 |  | 0.99 | 0.96, 1.03 | 0.922 |
| Q4 – Least deprived | 1.00 | 0.84, 1.18 | 0.967 |  | 1.01 | 0.89, 1.14 | 0.907 |  | 1.00 | 0.97, 1.03 | 0.958 |

Analyses were conducted using ordinal logistic regression for self-reported general health and quasi-Poisson regression for hospital episodes; complex survey weights were implemented to approximate the target population. Odds Ratios (OR), Incidence Rate Ratios (IRR) and their 95% confidence intervals (CI) are reported per 1 µg m^-3^ increment. Sample sizes are presented in the flowchart (see Figure 2).

All models were adjusted for age, sex, ethnic groups, number of siblings, highest household education, household tenure, household employment, and partnership status. Time-varying data on country (for self-reported general health only), area-level income deprivation and population density were included from the respective exposure period.

**Supplementary Figure 6:** Stratified analyses between air pollution, self-reported general health and number of hospital episodes, across (A) educational and (B-H) deprivation groups in the fully-adjusted models, Millennium Cohort Study. Analyses were conducted using ordinal logistic regression for self-reported general health and quasi-Poisson regression for hospital episodes; complex survey weights were implemented to approximate the target population. All models were adjusted for age, sex, ethnic groups, number of siblings, highest household education, household tenure, household employment, and partnership status. Time-varying data on country (for self-reported general health only), area-level income deprivation and population density were included from the respective exposure period.

**A**: Self-reported general health and PM_2.5_, PM_10_, and NO_2_ exposure in infancy by highest household education


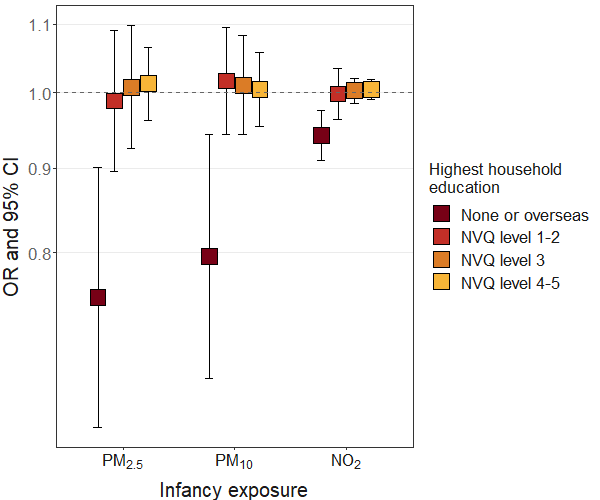


**B**. Self-reported general health and PM_2.5_ by area-level income deprivation


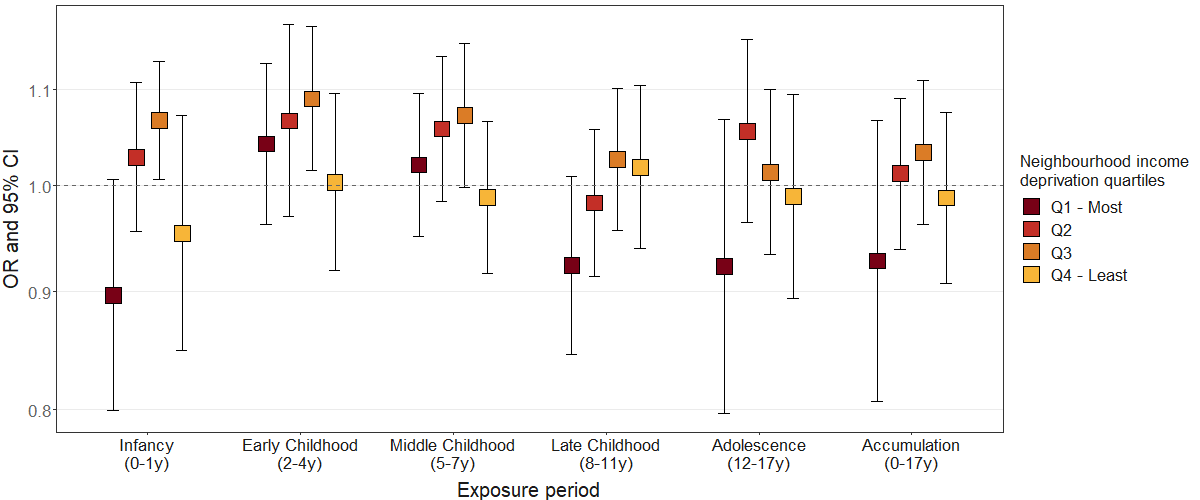


**D**. Self-reported general health and PM_10_ by area-level income deprivation


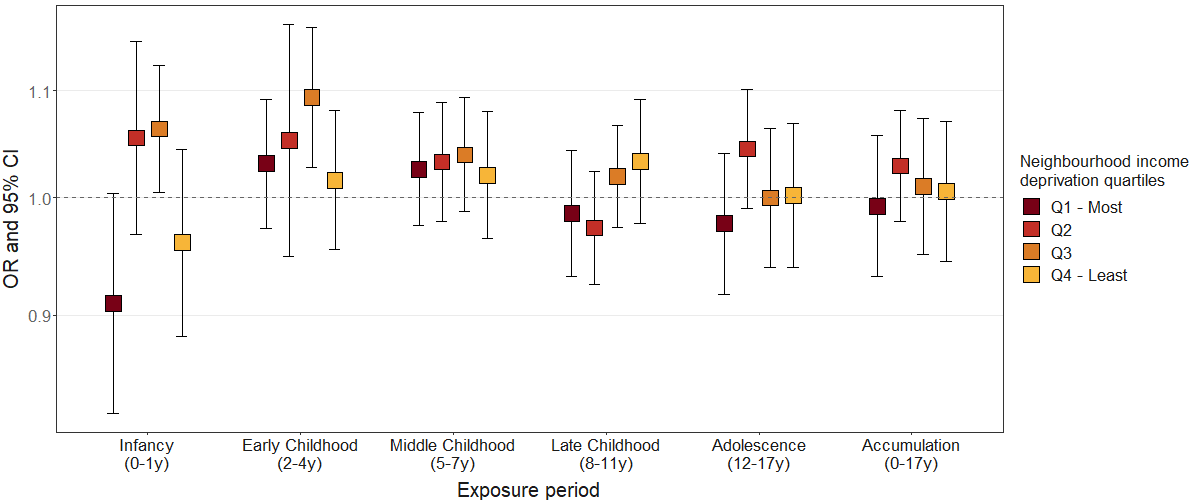


**E**. Self-reported general health and NO_2_ by area-level income deprivation

**
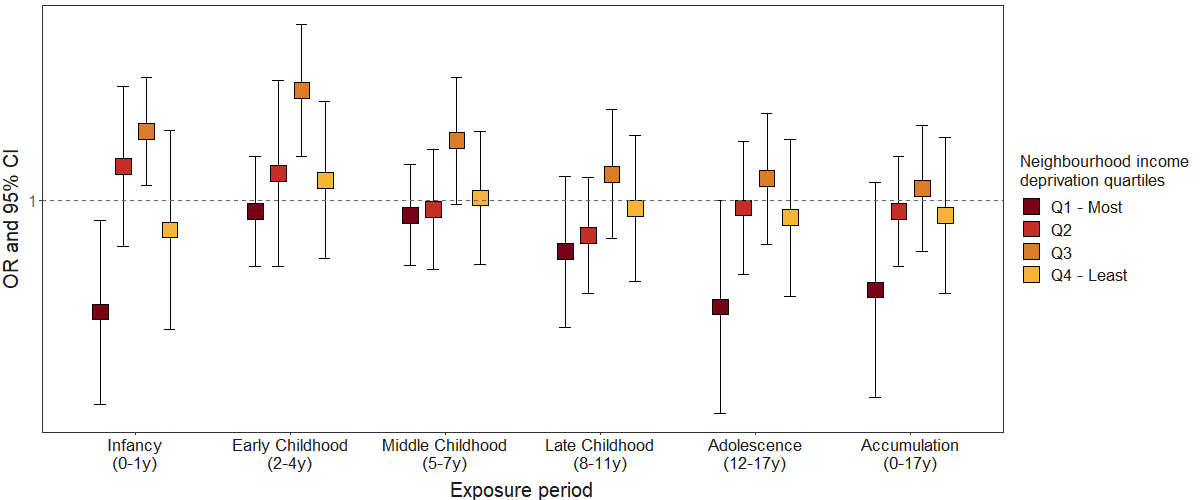
**

**F**. Number of hospital episodes and PM_2.5_ by area-level income deprivation


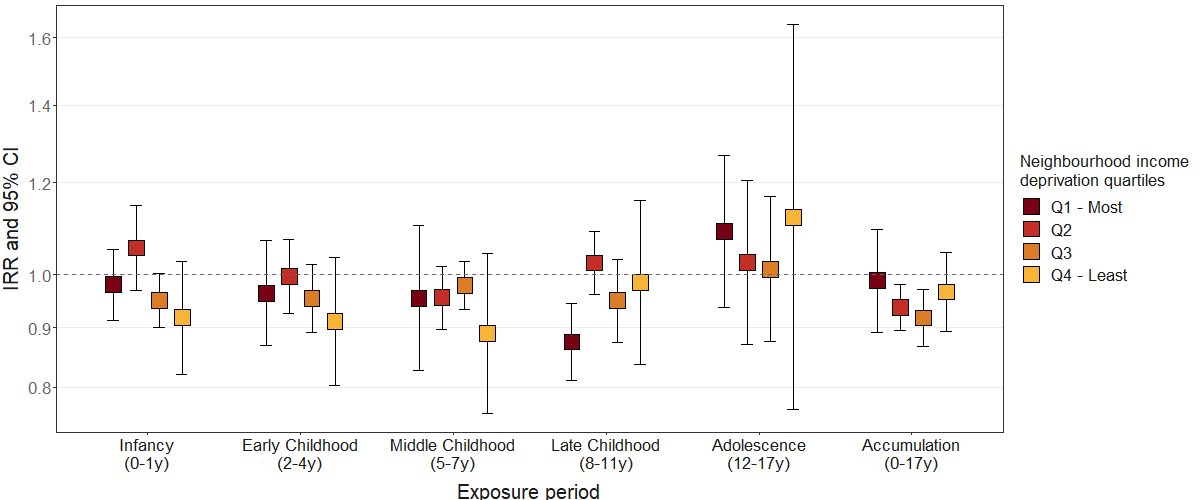


**G**. Number of hospital episodes and PM_10_ by area-level income deprivation


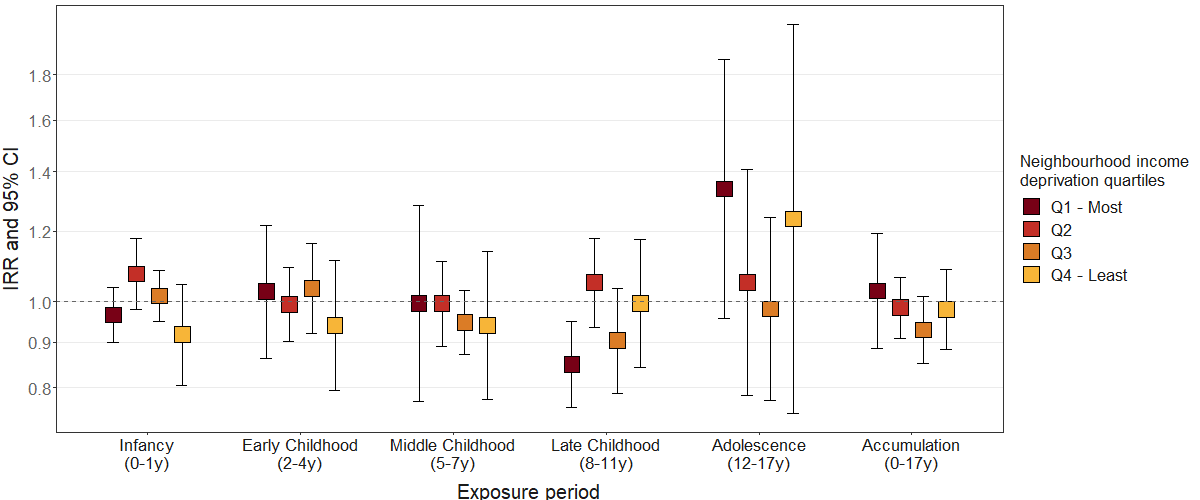


**H**. Number of hospital episodes and NO_2_ by area-level income deprivation

**
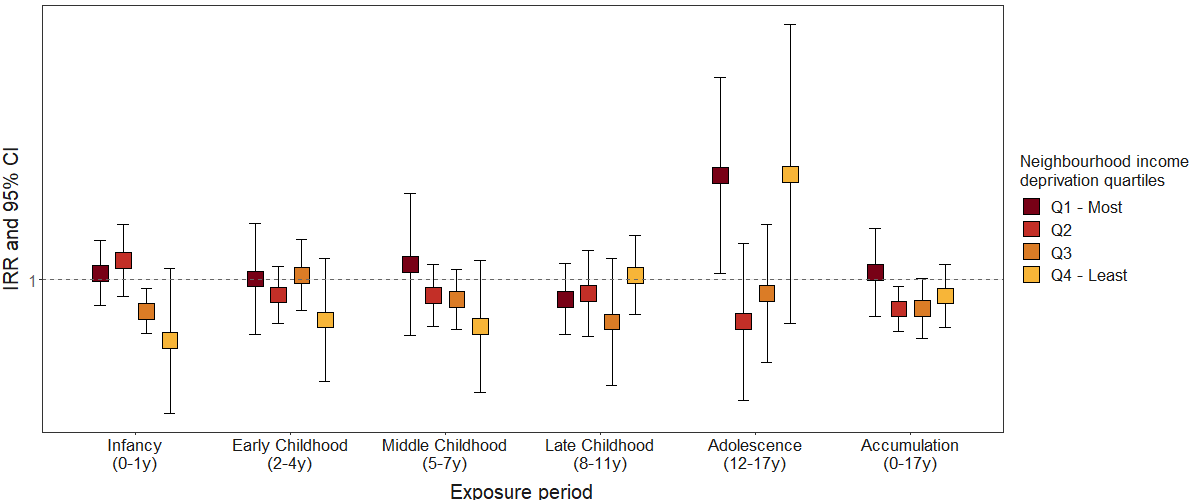
**

**Supplementary Table 12**: Air pollution exposure using 100m buffers, self-reported general health and number of hospital episodes, Millennium Cohort Study.

|  | **Model 1** | | |  | **Model 2** | | |  | **Model 3** | | |
| --- | --- | --- | --- | --- | --- | --- | --- | --- | --- | --- | --- |
| **(A) Self-reported general health^a^** | OR | 95% CI | *p* |  | OR | 95% CI | *p* |  | OR | 95% CI | *p* |
| **PM_2.5_** |  |  |  |  |  |  |  |  |  |  |  |
| Infancy | 1.02 | 0.99, 1.06 | 0.176 |  | 1.00 | 0.96, 1.04 | 0.979 |  | 0.98 | 0.92, 1.03 | 0.386 |
| Early Childhood | **1.08** | **1.05, 1.11** | **<0.001** |  | **1.06** | **1.03, 1.09** | **<0.001** |  | **1.05** | **1.01, 1.10** | **0.021** |
| Middle Childhood | **1.05** | **1.03, 1.07** | **<0.001** |  | **1.03** | **1.01, 1.05** | **0.006** |  | **1.03** | **1.00, 1.07** | **0.049** |
| Late Childhood | **1.03** | **1.01, 1.06** | **0.012** |  | 1.01 | 0.99, 1.04 | 0.320 |  | 0.99 | 0.95, 1.03 | 0.518 |
| Adolescence | **1.05** | **1.01, 1.08** | **0.010** |  | 1.02 | 0.97, 1.06 | 0.466 |  | 0.99 | 0.94, 1.05 | 0.845 |
| Accumulation | **1.04** | **1.01, 1.08** | **0.015** |  | 1.02 | 0.98, 1.06 | 0.439 |  | 0.99 | 0.93, 1.04 | 0.600 |
| **PM_10_** | | | | | | | | | | | |
| Infancy | 1.02 | 0.99, 1.04 | 0.139 |  | 1.00 | 0.98, 1.03 | 0.828 |  | 0.98 | 0.94, 1.03 | 0.429 |
| Early Childhood | **1.05** | **1.03, 1.07** | **<0.001** |  | **1.04** | **1.02, 1.06** | **<0.001** |  | **1.04** | **1.01, 1.08** | **0.017** |
| Middle Childhood | **1.03** | **1.02, 1.04** | **<0.001** |  | **1.02** | **1.01, 1.04** | **0.002** |  | **1.03** | **1.00, 1.05** | **0.043** |
| Late Childhood | **1.03** | **1.01, 1.05** | **0.002** |  | 1.02 | 1.00, 1.04 | 0.111 |  | 1.00 | 0.98, 1.03 | 0.879 |
| Adolescence | **1.04** | **1.02, 1.06** | **<0.001** |  | 1.02 | 0.99, 1.04 | 0.141 |  | 1.00 | 0.97, 1.04 | 0.761 |
| Accumulation | **1.04** | **1.02, 1.06** | **<0.001** |  | 1.02 | 1.00, 1.04 | 0.062 |  | 1.01 | 0.97, 1.04 | 0.678 |
| **NO_2_** |  |  |  |  |  |  |  |  |  |  |  |
| Infancy | 1.00 | 1.00, 1.01 | 0.213 |  | 1.00 | 0.99, 1.01 | 0.537 |  | 0.99 | 0.98, 1.01 | 0.267 |
| Early Childhood | **1.02** | **1.01, 1.02** | **<0.001** |  | **1.01** | **1.00, 1.02** | **0.001** |  | **1.01** | **1.00, 1.02** | **0.042** |
| Middle Childhood | **1.01** | **1.01, 1.02** | **<0.001** |  | 1.00 | 1.00, 1.01 | 0.090 |  | 1.00 | 0.99, 1.01 | 0.751 |
| Late Childhood | **1.01** | **1.00, 1.02** | **0.002** |  | 1.00 | 0.99, 1.01 | 0.566 |  | 0.99 | 0.99, 1.00 | 0.293 |
| Adolescence | **1.01** | **1.00, 1.02** | **0.010** |  | 1.00 | 0.99, 1.01 | 0.903 |  | 0.99 | 0.98, 1.00 | 0.141 |
| Accumulation | **1.01** | **1.00, 1.02** | **0.038** |  | 1.00 | 0.99, 1.01 | 0.856 |  | 0.99 | 0.98, 1.00 | 0.156 |
| **(B) Number of hospital episodes^b^** | IRR | 95% CI | *p* |  | IRR | 95% CI | *p* |  | IRR | 95% CI | *p* |
| **PM_2.5_** | | | | | | | | | | | |
| Infancy | 1.01 | 0.97, 1.06 | 0.556 |  | 1.00 | 0.96, 1.04 | 0.951 |  | 0.99 | 0.95, 1.03 | 0.570 |
| Early Childhood | 1.02 | 0.95, 1.11 | 0.571 |  | 1.01 | 0.93, 1.10 | 0.793 |  | 0.99 | 0.92, 1.06 | 0.777 |
| Middle Childhood | 1.01 | 0.93, 1.10 | 0.798 |  | 1.01 | 0.92, 1.11 | 0.845 |  | 0.97 | 0.90, 1.05 | 0.454 |
| Late Childhood | 0.97 | 0.91, 1.03 | 0.341 |  | 0.96 | 0.90, 1.03 | 0.265 |  | 0.95 | 0.89, 1.03 | 0.210 |
| Adolescence | 1.19 | 0.99, 1.44 | 0.064 |  | **1.22** | **1.02, 1.45** | **0.031** |  | **1.27** | **1.03, 1.55** | **0.024** |
| Accumulation | 1.02 | 0.96, 1.09 | 0.454 |  | 1.02 | 0.96, 1.09 | 0.493 |  | 1.00 | 0.95, 1.06 | 0.980 |
| **PM_10_** | | | | | | | | | | | |
| Infancy | 0.99 | 0.96, 1.03 | 0.713 |  | 0.99 | 0.95, 1.03 | 0.546 |  | 0.98 | 0.95, 1.02 | 0.303 |
| Early Childhood | 0.99 | 0.94, 1.03 | 0.511 |  | 0.98 | 0.93, 1.03 | 0.508 |  | 0.96 | 0.92, 1.01 | 0.107 |
| Middle Childhood | 0.99 | 0.94, 1.04 | 0.638 |  | 0.99 | 0.93, 1.05 | 0.713 |  | 0.96 | 0.91, 1.01 | 0.110 |
| Late Childhood | 0.96 | 0.91, 1.02 | 0.176 |  | 0.96 | 0.91, 1.01 | 0.111 |  | 0.95 | 0.90, 1.01 | 0.090 |
| Adolescence | 1.07 | 0.98, 1.17 | 0.128 |  | 1.09 | 0.99, 1.19 | 0.078 |  | 1.12 | 0.99, 1.28 | 0.069 |
| Accumulation | 0.99 | 0.95, 1.02 | 0.528 |  | 0.99 | 0.95, 1.03 | 0.607 |  | 0.97 | 0.94, 1.01 | 0.111 |
| **NO_2_** |  |  |  |  |  |  |  |  |  |  |  |
| Infancy | 1.01 | 0.99, 1.03 | 0.558 |  | 1.00 | 0.99, 1.02 | 0.648 |  | 1.00 | 0.98, 1.02 | 0.705 |
| Early Childhood | 1.00 | 0.99, 1.01 | 0.822 |  | 1.00 | 0.98, 1.02 | 0.959 |  | 0.99 | 0.98, 1.01 | 0.277 |
| Middle Childhood | 1.01 | 0.99, 1.02 | 0.531 |  | 1.01 | 0.98, 1.03 | 0.588 |  | 0.99 | 0.98, 1.01 | 0.389 |
| Late Childhood | 1.01 | 0.99, 1.03 | 0.418 |  | 1.00 | 0.99, 1.01 | 0.932 |  | 1.00 | 0.98, 1.01 | 0.804 |
| Adolescence | 1.03 | 0.99, 1.06 | 0.125 |  | **1.04** | **1.00, 1.07** | **0.047** |  | **1.05** | **1.00, 1.10** | **0.039** |
| Accumulation | 1.00 | 0.99, 1.01 | 0.798 |  | 1.00 | 0.99, 1.02 | 0.851 |  | 0.99 | 0.98, 1.01 | 0.298 |

Analyses were conducted using ordinal logistic regression for self-reported general health and quasi-Poisson regression for hospital episodes; complex survey weights were implemented to approximate the target population. Odds Ratios (OR), Incidence Rate Ratios (IRR) and their 95% confidence intervals (CI) are reported per 1 µg m^-3^ increment. Sample sizes are presented in the flowchart (see Figure 2).

Model 1: adjusted for age and sex.

Model 2: Model 1 + ethnic groups, number of siblings, highest household education, household tenure, household employment, and partnership status (time-invariant).

Model 3: Model 2 + country (for self-reported general health only), area-level income deprivation and population density (time-varying).

^a^ United Kingdom

^b^ England

**Supplementary Table 13**: Air pollution exposure using 500m buffers, self-reported general health and number of hospital episodes, Millennium Cohort Study.

|  | **Model 1** | | |  | **Model 2** | | |  | **Model 3** | | |
| --- | --- | --- | --- | --- | --- | --- | --- | --- | --- | --- | --- |
| **(A) Self-reported general health^a^** | OR | 95% CI | *p* |  | OR | 95% CI | *p* |  | OR | 95% CI | *p* |
| **PM_2.5_** |  |  |  |  |  |  |  |  |  |  |  |
| Infancy | 1.02 | 0.99, 1.06 | 0.173 |  | 1.00 | 0.96, 1.04 | 0.977 |  | 0.98 | 0.92, 1.03 | 0.380 |
| Early Childhood | **1.08** | **1.05, 1.11** | **<0.001** |  | **1.06** | **1.03, 1.09** | **<0.001** |  | **1.05** | **1.01, 1.10** | **0.023** |
| Middle Childhood | **1.05** | **1.03, 1.07** | **<0.001** |  | **1.03** | **1.01, 1.06** | **0.004** |  | **1.04** | **1.00, 1.07** | **0.032** |
| Late Childhood | **1.03** | **1.01, 1.06** | **0.007** |  | 1.02 | 0.99, 1.04 | 0.264 |  | 0.99 | 0.95, 1.03 | 0.617 |
| Adolescence | **1.05** | **1.01, 1.08** | **0.008** |  | 1.01 | 0.97, 1.06 | 0.490 |  | 0.99 | 0.94, 1.05 | 0.784 |
| Accumulation | **1.05** | **1.01, 1.08** | **0.006** |  | 1.02 | 0.98, 1.06 | 0.334 |  | 0.99 | 0.94, 1.04 | 0.730 |
| **PM_10_** | | | | | | | | | | | |
| Infancy | 1.02 | 1.00, 1.05 | 0.080 |  | 1.01 | 0.98, 1.03 | 0.717 |  | 0.98 | 0.94, 1.03 | 0.500 |
| Early Childhood | **1.05** | **1.03, 1.07** | **<0.001** |  | **1.04** | **1.02, 1.07** | **<0.001** |  | **1.05** | **1.01, 1.09** | **0.010** |
| Middle Childhood | **1.03** | **1.02, 1.05** | **<0.001** |  | **1.03** | **1.01, 1.04** | **0.001** |  | **1.03** | **1.01, 1.06** | **0.015** |
| Late Childhood | **1.03** | **1.01, 1.05** | **0.001** |  | 1.02 | 1.00, 1.04 | 0.092 |  | 1.00 | 0.98, 1.03 | 0.849 |
| Adolescence | **1.04** | **1.02, 1.06** | **<0.001** |  | 1.02 | 1.00, 1.04 | 0.118 |  | 1.01 | 0.97, 1.04 | 0.740 |
| Accumulation | **1.04** | **1.02, 1.06** | **<0.001** |  | **1.02** | **1.00, 1.05** | **0.037** |  | 1.01 | 0.98, 1.04 | 0.529 |
| **NO_2_** |  |  |  |  |  |  |  |  |  |  |  |
| Infancy | 1.01 | 1.00, 1.01 | 0.127 |  | 1.00 | 0.99, 1.01 | 0.657 |  | 0.99 | 0.98, 1.01 | 0.349 |
| Early Childhood | **1.02** | **1.01, 1.02** | **<0.001** |  | **1.01** | **1.00, 1.02** | **0.002** |  | 1.01 | 1.00, 1.02 | 0.067 |
| Middle Childhood | **1.01** | **1.01, 1.02** | **<0.001** |  | 1.01 | 1.00, 1.01 | 0.054 |  | 1.00 | 1.00, 1.01 | 0.539 |
| Late Childhood | **1.01** | **1.00, 1.02** | **<0.001** |  | 1.00 | 0.99, 1.01 | 0.539 |  | 0.99 | 0.99, 1.00 | 0.236 |
| Adolescence | **1.01** | **1.00, 1.02** | **0.007** |  | 1.00 | 0.99, 1.01 | 0.843 |  | 0.99 | 0.98, 1.00 | 0.073 |
| Accumulation | **1.01** | **1.00, 1.02** | **0.013** |  | 1.00 | 0.99, 1.01 | 0.999 |  | 0.99 | 0.98, 1.00 | 0.185 |
| **(B) Number of hospital episodes^b^** | IRR | 95% CI | *p* |  | IRR | 95% CI | *p* |  | IRR | 95% CI | *p* |
| **PM_2.5_** | | | | | | | | | | | |
| Infancy | 1.00 | 0.96, 1.04 | 0.889 |  | 0.99 | 0.94, 1.03 | 0.541 |  | 0.98 | 0.93, 1.02 | 0.291 |
| Early Childhood | 1.03 | 0.94, 1.12 | 0.497 |  | 1.02 | 0.93, 1.12 | 0.690 |  | 1.00 | 0.92, 1.08 | 0.948 |
| Middle Childhood | 1.02 | 0.92, 1.12 | 0.711 |  | 1.02 | 0.91, 1.14 | 0.757 |  | 0.98 | 0.90, 1.07 | 0.637 |
| Late Childhood | 0.94 | 0.87, 1.02 | 0.165 |  | 0.94 | 0.87, 1.01 | 0.099 |  | 0.93 | 0.86, 1.00 | 0.063 |
| Adolescence | 1.17 | 0.96, 1.42 | 0.115 |  | 1.20 | 1.00, 1.46 | 0.056 |  | **1.26** | **1.00, 1.58** | **0.047** |
| Accumulation | 1.03 | 0.96, 1.10 | 0.426 |  | 1.03 | 0.96, 1.11 | 0.459 |  | 1.01 | 0.95, 1.07 | 0.853 |
| **PM_10_** | | | | | | | | | | | |
| Infancy | 1.00 | 0.96, 1.05 | 0.988 |  | 1.00 | 0.95, 1.05 | 0.954 |  | 0.99 | 0.95, 1.04 | 0.721 |
| Early Childhood | 0.99 | 0.94, 1.04 | 0.646 |  | 0.99 | 0.93, 1.05 | 0.630 |  | 0.96 | 0.91, 1.01 | 0.153 |
| Middle Childhood | 0.99 | 0.93, 1.05 | 0.761 |  | 0.99 | 0.92, 1.07 | 0.820 |  | 0.96 | 0.91, 1.02 | 0.158 |
| Late Childhood | 0.96 | 0.91, 1.01 | 0.147 |  | 0.96 | 0.91, 1.01 | 0.098 |  | 0.95 | 0.90, 1.01 | 0.075 |
| Adolescence | 1.05 | 0.96, 1.15 | 0.262 |  | 1.07 | 0.98, 1.17 | 0.157 |  | 1.10 | 0.97, 1.25 | 0.129 |
| Accumulation | 0.99 | 0.95, 1.03 | 0.577 |  | 0.99 | 0.94, 1.04 | 0.669 |  | 0.97 | 0.93, 1.01 | 0.133 |
| **NO_2_** |  |  |  |  |  |  |  |  |  |  |  |
| Infancy | 1.00 | 0.99, 1.01 | 0.844 |  | 1.00 | 0.98, 1.01 | 0.611 |  | 0.99 | 0.98, 1.01 | 0.306 |
| Early Childhood | 1.00 | 0.99, 1.02 | 0.649 |  | 1.00 | 0.98, 1.02 | 0.778 |  | 1.00 | 0.98, 1.01 | 0.613 |
| Middle Childhood | 1.01 | 0.99, 1.03 | 0.430 |  | 1.01 | 0.98, 1.03 | 0.485 |  | 1.00 | 0.98, 1.02 | 0.776 |
| Late Childhood | 1.00 | 0.99, 1.01 | 0.831 |  | 0.99 | 0.98, 1.01 | 0.370 |  | 0.99 | 0.97, 1.00 | 0.118 |
| Adolescence | 1.03 | 0.99, 1.06 | 0.156 |  | 1.04 | 1.00, 1.08 | 0.066 |  | 1.05 | 1.00, 1.11 | 0.061 |
| Accumulation | 1.00 | 0.99, 1.01 | 0.727 |  | 1.00 | 0.99, 1.02 | 0.750 |  | 1.00 | 0.98, 1.01 | 0.509 |

Analyses were conducted using ordinal logistic regression for self-reported general health and quasi-Poisson regression for hospital episodes; complex survey weights were implemented to approximate the target population. Odds Ratios (OR), Incidence Rate Ratios (IRR) and their 95% confidence intervals (CI) are reported per 1 µg m^-3^ increment. Sample sizes are presented in the flowchart (see Figure 2).

Model 1: adjusted for age and sex.

Model 2: Model 1 + ethnic groups, number of siblings, highest household education, household tenure, household employment, and partnership status (time-invariant).

Model 3: Model 2 + country (for self-reported general health only), area-level income deprivation and population density (time-varying).

^a^ United Kingdom

^b^ England

**Supplementary Table 14**: Air pollution exposure from birth onwards, self-reported general health and number of hospital episodes after adjusting for maternal health, Millennium Cohort Study.

|  | **PM_2.5_** | | |  | **PM_10_** | | |  | **NO_2_** | | |
| --- | --- | --- | --- | --- | --- | --- | --- | --- | --- | --- | --- |
| Self-reported general health | OR | 95% CI | *p* |  | OR | 95% CI | *p* |  | OR | 95% CI | *p* |
| Infancy (n=9593) | 0.96 | 0.91, 1.02 | 0.190 |  | 0.98 | 0.93, 1.02 | 0.355 |  | 0.99 | 0.98, 1.01 | 0.450 |
| Early Childhood (n=9137) | 1.05 | 1.00, 1.10 | 0.060 |  | **1.05** | **1.01, 1.08** | **0.018** |  | **1.01** | **1.00, 1.02** | **0.021** |
| Middle Childhood (n=9171) | **1.04** | **1.00, 1.07** | **0.041** |  | **1.03** | **1.01, 1.06** | **0.019** |  | 1.00 | 0.99, 1.01 | 0.807 |
| Late Childhood (n=9422) | 1.00 | 0.96, 1.04 | 0.860 |  | 1.01 | 0.98, 1.04 | 0.500 |  | 1.00 | 0.99, 1.01 | 0.446 |
| Adolescence (n=9216) | 0.99 | 0.93, 1.05 | 0.724 |  | 1.01 | 0.98, 1.04 | 0.537 |  | 0.99 | 0.98, 1.00 | 0.082 |
| Accumulation (n=9216) | 0.98 | 0.93, 1.03 | 0.421 |  | 1.01 | 0.98, 1.04 | 0.557 |  | 0.99 | 0.98, 1.00 | 0.120 |
| Number of hospital episodes | IRR | 95% CI | *p* |  | IRR | 95% CI | *p* |  | IRR | 95% CI | *p* |
| Infancy (n=5269) | 0.98 | 0.94, 1.02 | 0.291 |  | 0.97 | 0.94, 1.01 | 0.147 |  | 0.99 | 0.98, 1.01 | 0.314 |
| Early Childhood (n=4923) | 0.98 | 0.92, 1.06 | 0.663 |  | **0.95** | **0.91, 1.00** | **0.041** |  | 0.99 | 0.98, 1.01 | 0.274 |
| Middle Childhood (n=4886) | 0.98 | 0.90, 1.06 | 0.580 |  | 0.95 | 0.90, 1.00 | 0.068 |  | 0.99 | 0.98, 1.01 | 0.406 |
| Late Childhood (n=5059) | 0.96 | 0.89, 1.04 | 0.283 |  | 0.96 | 0.91, 1.01 | 0.138 |  | 0.99 | 0.98, 1.01 | 0.537 |
| Adolescence (n=5043) | **1.32** | **1.06, 1.64** | **0.014** |  | 1.13 | 0.99, 1.28 | 0.068 |  | **1.06** | **1.01, 1.11** | **0.018** |
| Accumulation (n=5043) | 0.99 | 0.95, 1.04 | 0.830 |  | **0.97** | **0.94, 1.00** | **0.044** |  | 0.99 | 0.98, 1.01 | 0.324 |

Analyses were conducted using ordinal logistic regression for self-reported general health and quasi-Poisson regression for hospital episodes; complex survey weights were implemented to approximate the target population. Odds Ratios (OR), Incidence Rate Ratios (IRR) and their 95% confidence intervals (CI) are reported per 1 µg m^-3^ increment.

All models were adjusted for age, sex, ethnic groups, number of siblings, highest household education, household tenure, household employment, and partnership status. Time-varying data on country (for self-reported general health only), area-level income deprivation and population density were included from the respective exposure period. Models were further adjusted for maternal self-reported general health, maternal BMI before birth and maternal smoking.

**Supplementary Table 15**: Air pollution exposure from birth onwards, self-reported general health and number of hospital episodes after adjusting for road traffic noise, Millennium Cohort Study.

|  | **PM_2.5_** | | |  | **PM_10_** | | |  | **NO_2_** | | |
| --- | --- | --- | --- | --- | --- | --- | --- | --- | --- | --- | --- |
| Self-reported general health | OR | 95% CI | *p_FDR_* |  | OR | 95% CI | *p_FDR_* |  | OR | 95% CI | *p_FDR_* |
| Infancy | 0.97 | 0.92, 1.03 | 0.290 |  | 0.98 | 0.94, 1.03 | 0.444 |  | 0.99 | 0.98, 1.01 | 0.207 |
| Early Childhood | **1.05** | **1.00, 1.10** | **0.036** |  | **1.05** | **1.01, 1.09** | **0.014** |  | 1.01 | 1.00, 1.02 | 0.070 |
| Middle Childhood | 1.03 | 1.00, 1.07 | 0.059 |  | **1.03** | **1.00, 1.05** | **0.024** |  | 1.00 | 0.99, 1.01 | 0.729 |
| Late Childhood | 0.99 | 0.95, 1.03 | 0.481 |  | 1.00 | 0.98, 1.03 | 0.846 |  | 0.99 | 0.99, 1.00 | 0.249 |
| Adolescence | 0.99 | 0.93, 1.05 | 0.636 |  | 1.00 | 0.97, 1.04 | 0.821 |  | 0.99 | 0.98, 1.00 | 0.072 |
| Accumulation | 0.98 | 0.93, 1.04 | 0.478 |  | 1.01 | 0.97, 1.04 | 0.684 |  | 0.99 | 0.98, 1.00 | 0.117 |
| Number of hospital episodes | IRR | 95% CI | *p_FDR_* |  | IRR | 95% CI | *p_FDR_* |  | IRR | 95% CI | *p_FDR_* |
| Infancy | 0.98 | 0.94, 1.02 | 0.393 |  | 0.98 | 0.94, 1.03 | 0.444 |  | 1.00 | 0.98, 1.02 | 0.980 |
| Early Childhood | 1.00 | 0.92, 1.07 | 0.935 |  | 0.96 | 0.92, 1.01 | 0.081 |  | 0.99 | 0.98, 1.01 | 0.357 |
| Middle Childhood | 0.97 | 0.90, 1.06 | 0.502 |  | 0.95 | 0.90, 1.00 | 0.057 |  | 0.99 | 0.98, 1.01 | 0.374 |
| Late Childhood | 0.94 | 0.87, 1.01 | 0.097 |  | 0.95 | 0.89, 1.00 | 0.051 |  | 0.99 | 0.98, 1.01 | 0.412 |
| Adolescence | 1.22 | 0.99, 1.50 | 0.064 |  | 1.09 | 0.97, 1.23 | 0.155 |  | 1.04 | 1.00, 1.09 | 0.079 |
| Accumulation | 0.99 | 0.93, 1.05 | 0.675 |  | **0.96** | **0.92, 1.00** | **0.028** |  | 0.99 | 0.98, 1.00 | 0.163 |

Analyses were conducted using ordinal logistic regression for self-reported general health and quasi-Poisson regression for hospital episodes; complex survey weights were implemented to approximate the target population. Odds Ratios (OR), Incidence Rate Ratios (IRR) and their 95% confidence intervals (CI) are reported per 1 µg m^-3^ increment. Sample sizes are presented in the flowchart (see Figure 2).

All models were adjusted for age, sex, ethnic groups, number of siblings, highest household education, household tenure, household employment, and partnership status. Time-varying data on country (for self-reported general health only), area-level income deprivation, road traffic noise and population density were included from the respective exposure period.

**Supplementary Table 16**: Air pollution exposure from birth onwards, self-reported general health and number of hospital episodes after multiple imputation in the fully-adjusted models, Millennium Cohort Study.

|  | **PM_2.5_** | | |  | **PM_10_** | | |  | **NO_2_** | | |
| --- | --- | --- | --- | --- | --- | --- | --- | --- | --- | --- | --- |
| Self-reported general health  (n=9971)^a^ | OR | 95% CI | *p* |  | OR | 95% CI | *p* |  | OR | 95% CI | *p* |
| Infancy | 0.98 | 0.92, 1.03 | 0.380 |  | 0.98 | 0.94, 1.03 | 0.500 |  | 0.99 | 0.98, 1.01 | 0.294 |
| Early Childhood | **1.06** | **1.01, 1.11** | **0.016** |  | **1.05** | **1.01, 1.09** | **0.010** |  | **1.01** | **1.00, 1.02** | **0.037** |
| Middle Childhood | **1.04** | **1.00, 1.07** | **0.038** |  | **1.03** | **1.00, 1.06** | **0.022** |  | 1.00 | 1.00, 1.01 | 0.579 |
| Late Childhood | 0.99 | 0.95, 1.03 | 0.510 |  | 1.00 | 0.98, 1.03 | 0.854 |  | 1.00 | 0.99, 1.00 | 0.277 |
| Adolescence | 0.99 | 0.94, 1.05 | 0.844 |  | 1.01 | 0.98, 1.04 | 0.714 |  | 0.99 | 0.98, 1.00 | 0.131 |
| Accumulation | 0.99 | 0.94, 1.04 | 0.646 |  | 1.01 | 0.98, 1.04 | 0.583 |  | 0.99 | 0.98, 1.00 | 0.173 |
| Infancy | 0.98 | 0.92, 1.03 | 0.380 |  | 0.98 | 0.94, 1.03 | 0.500 |  | 0.99 | 0.98, 1.01 | 0.294 |
| Number of hospital episodes  (n=6104)^b^ | IRR | 95% CI | *p* |  | IRR | 95% CI | *p* |  | IRR | 95% CI | *p* |
| Infancy | 0.98 | 0.95, 1.02 | 0.421 |  | 0.99 | 0.95, 1.03 | 0.597 |  | 1.00 | 0.98, 1.02 | 0.961 |
| Early Childhood | 0.98 | 0.92, 1.05 | 0.641 |  | 0.96 | 0.91, 1.00 | 0.070 |  | 0.99 | 0.98, 1.01 | 0.320 |
| Middle Childhood | 0.97 | 0.89, 1.05 | 0.419 |  | 0.95 | 0.90, 1.00 | 0.070 |  | 0.99 | 0.98, 1.01 | 0.464 |
| Late Childhood | 0.94 | 0.87, 1.02 | 0.117 |  | 0.95 | 0.90, 1.00 | 0.072 |  | 1.00 | 0.98, 1.01 | 0.561 |
| Adolescence | **1.27** | **1.04, 1.55** | **0.017** |  | 1.11 | 0.99, 1.26 | 0.075 |  | **1.05** | **1.01, 1.10** | **0.018** |
| Accumulation | 1.00 | 0.95, 1.05 | 0.941 |  | 0.97 | 0.93, 1.00 | 0.093 |  | 0.99 | 0.98, 1.00 | 0.278 |

Analyses were conducted using ordinal logistic regression for self-reported general health and quasi-Poisson regression for hospital episodes; complex survey weights were implemented to approximate the target population. Odds Ratios (OR), Incidence Rate Ratios (IRR) and their 95% confidence intervals (CI) are reported per 1 µg m^-3^ increment.

All models were adjusted for age, sex, ethnic groups, number of siblings, highest household education, household tenure, household employment, and partnership status. Time-varying data on country (for self-reported general health only), area-level income deprivation and population density were included from the respective exposure period. Missing data was imputed by multiple imputations on 25 datasets and coefficients were combined using Rubin’s rule.

^a^ United Kingdom

^b^ Lived in England and eligible for HES-APC data linkage.

**Supplementary Figure 7**: Multinomial regression showing the odds of reporting ‘very good’, ‘good’ and ‘fair or poor’ general health at age 17 by higher PM_2.5_, PM_10_ and NO_2_ exposure from birth onwards, Millennium Cohort Study. All models were adjusted for age, sex, ethnic groups, number of siblings, highest household education, household tenure, household employment, and partnership status; time-varying data on country, area-level income deprivation and population density were included from the respective exposure period. Complex survey weights were implemented to approximate the target population. ORs and their 95% CIs are reported per 1 µg m^-3^ increment. Sample sizes are presented in the flowchart (see Figure 2).

PM_2.5_
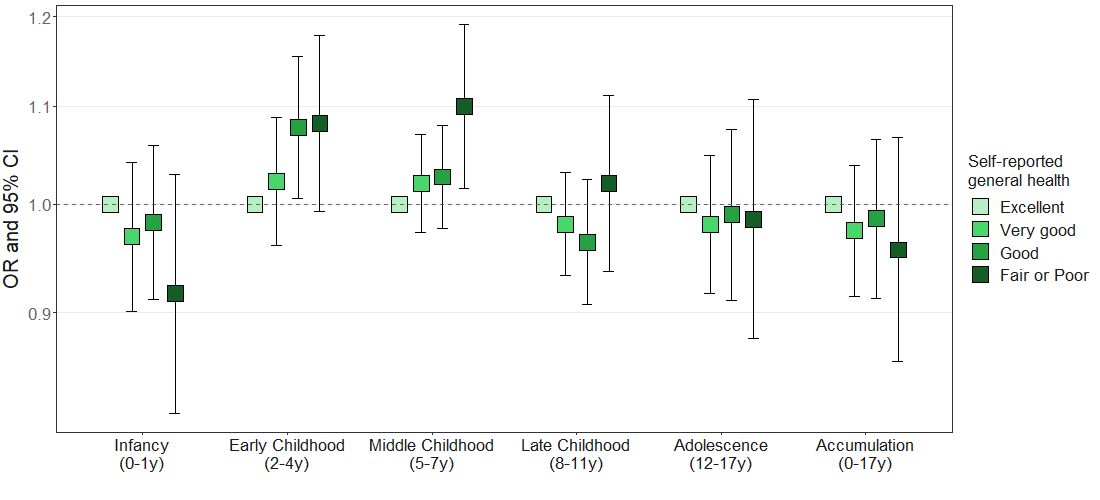


PM_10_
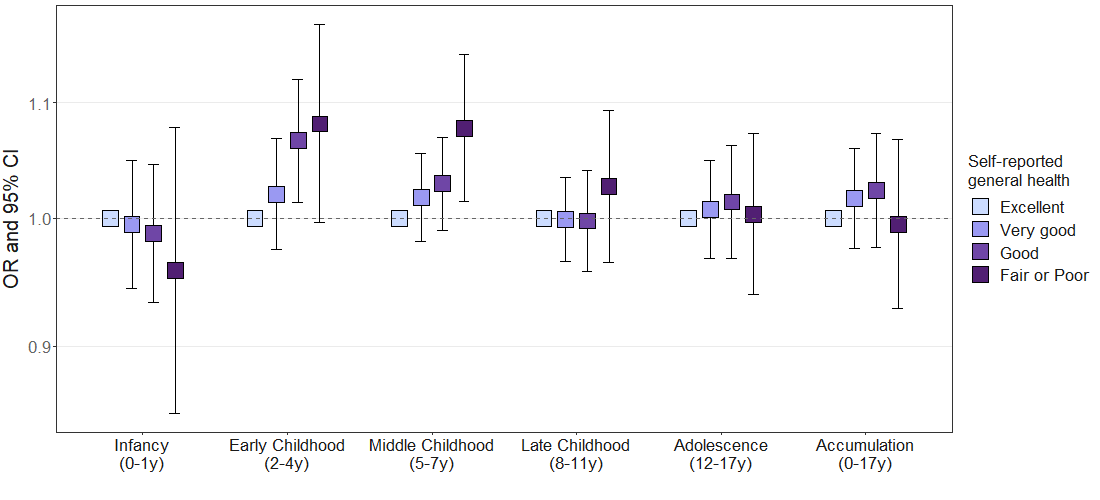


NO_2_


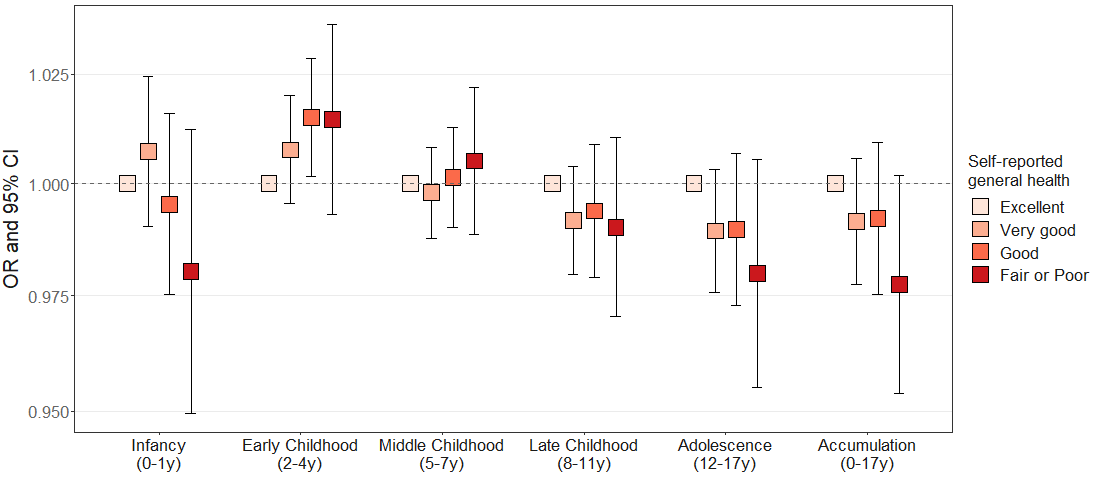


**Supplementary Figure 8**: Quantile g-computation for multinomial outcomes showing the odds of reporting ‘very good’, ‘good’ and ‘fair or poor’ general health at age 17 by higher air pollution exposure (mixture of PM_2.5_, PM_10_ and NO_2_) from birth onwards, Millennium Cohort Study. All models were adjusted for age, sex, ethnic groups, number of siblings, highest household education, household tenure, household employment, and partnership status; time-varying data on country, area-level income deprivation and population density were included from the respective exposure period. Survey weights were implemented to approximate the target population. ORs and their 95% CIs are reported per 1 µg m^-3^ increment. Sample sizes are presented in the flowchart (see Figure 2).


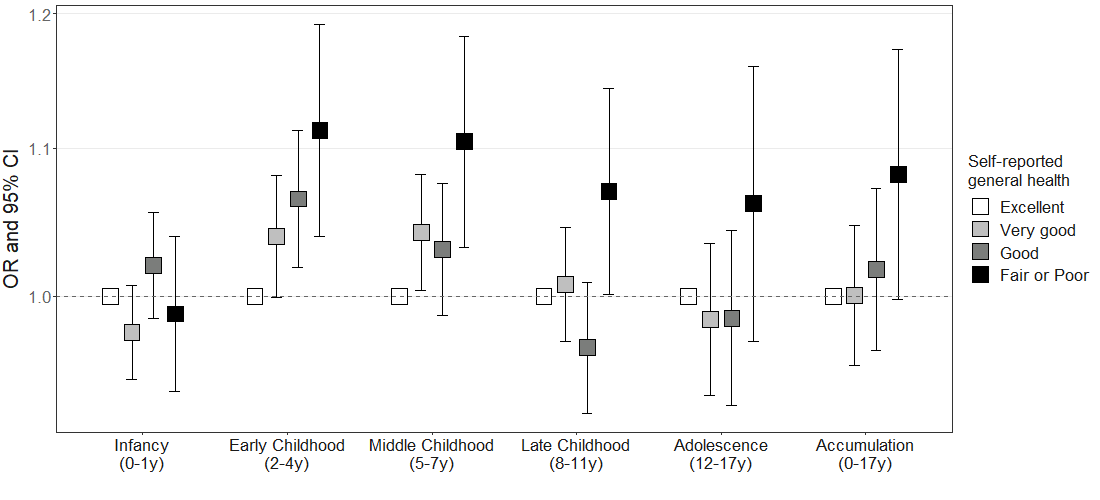

Supplement: Supplementary file 1 — Supplementary Material 1 [file 41598_2025_94107_MOESM1_ESM.docx]
